# Chimera patterns in conservative Hamiltonian systems and Bose–Einstein condensates of ultracold atoms

**DOI:** 10.1038/s41598-023-35061-3

**Published:** 2023-05-26

**Authors:** Hon Wai Hana Lau, Jörn Davidsen, Christoph Simon

**Affiliations:** 1 Institute for Quantum Science and Technology and Department of Physics and Astronomy, University of Calgary, Calgary, AB T2N 1N4 Canada; 2 Complexity Science Group, Department of Physics and Astronomy, University of Calgary, Calgary, T2N 1N4 Canada; 3 Hotchkiss Brain Institute, University of Calgary, Calgary, T2N 4N1 Canada; 4 Present Address: Quantum Information Science and Technology Unit, Okinawa Institute of Science and Technology Graduate University, Onna-son, Kunigami-gun, Okinawa 904-0495 Japan

**Keywords:** Statistical physics, thermodynamics and nonlinear dynamics, Ultracold gases, Matter waves and particle beams, Bose-Einstein condensates

## Abstract

Experimental realizations of chimera patterns, characterized by coexisting regions of phase coherence and incoherence, have so far been achieved for non-conservative systems with dissipation and exclusively in classical settings. The possibility of observing chimera patterns in quantum systems has rarely been studied and it remains an open question if chimera patterns can exist in closed, or conservative quantum systems. Here, we tackle these challenges by first proposing a conservative Hamiltonian system with nonlocal hopping, where the energy is well-defined and conserved. We show explicitly that such a system can exhibit chimera patterns. Then we propose a physical mechanism for the nonlocal hopping by using an additional mediating channel. This leads us to propose a possible experimentally realizable quantum system based on a two-component Bose–Einstein condensate (BEC) with a spin-dependent optical lattice, where an untrapped component serves as the matter-wave mediating field. In this BEC system, nonlocal spatial hopping over tens of lattice sites can be achieved and simulations suggest that chimera patterns should be observable in certain parameter regimes.

## Introduction

Chimera patterns are characterized by the coexistence of spatially localized regions of phase coherence and phase incoherence, which spontaneously break the symmetry in systems with translational invariance^1–4^. These patterns were first identified^5–7^ in the study of the complex Ginzburg–Landau equation (CGLE)^8,9^ with nonlocal diffusive coupling. About a decade after the discovery, these patterns have been experimentally demonstrated in chemical, mechanical, optical, electronic, and opto-electronic systems^10–20^. Chimera patterns also arise in neuronal systems, which suggests these patterns may serve certain biological function^21,22^. Theoretical studies of chimera patterns have been conducted across a wide range of systems in natural science^1–4,23–30^, including exciton-polariton^31,32^, coupled-waveguide resonators^33^, and metamaterials^34^, to name a few in physical systems. Over years, the studies also expanded to various oscillators, connection topology, patterns, and physical properties, as well as different notions of chimera patterns^1–4,27,35,36^. So far, chimera patterns have been exclusively observed in experiments involving classical dissipative and non-conservative systems. Only limited studies of chimera patterns have been carried out in quantum systems. All of them are in open quantum system settings with driving and dissipation such as time crystals^37–39^. Therefore, it is not yet clear what closed systems and quantum systems might exhibit chimera patterns.

**Figure 1:**
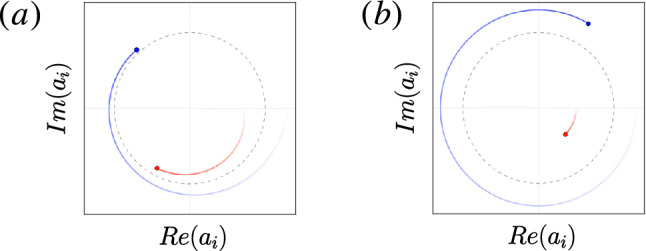
Illustration of the dynamics of two different types of oscillators in a two-dimensional phase space. (**a**) A self-sustained oscillator with a limit cycle attractor. Trajectories near a limit cycle (represented by the dotted unit circle) move toward it. Energy disspation and driving are present such that two different initial states tend to the same asymptotic dynamics with the same oscillation frequency as time goes to infinity. (**b**) A conservative nonlinear oscillator. Typically, the oscillation frequency depends on the initial condition. Since energy is conserved, trajectories corresponding to different initial energies remain separated at all times.

Here, we explore the existence of chimera patterns in conservative systems and quantum systems using a Hamiltonian approach. In classical physics, a system and its dynamics can be fully defined by specifying the total energy of the system in terms of the system parameters, called Hamiltonian^40^. A closed conservative system can be specified by a time independent Hamiltonian with constant energy. With such a Hamiltonian, there is a straightforward method to generalize to quantum systems by using a known quantization rule ansatz. The specific Hamiltonian systems we consider here are the multi-component Bose–Einstein condensates (BECs)^41–44^, which have a corresponding set of mean-field dynamic equations called Gross-Pitaevskii equations (GPEs)^45–47^. The one component GPE may be considered as a special case of the CGLE in certain limits and with some extensions^8,48^, so both possess global phase symmetry and third-order nonlinearity. Historically, the CGLE corresponds to the normal form of any spatially extended system close to a Hopf bifurcation—a critical point where a stationary system begins to oscillate^9,49^, and describes many physical systems phenomenologically, such as nonlinear waves^8,50^. Unlike the typical regime of the CGLE, the GPE locally behaves as an undamped non-linear oscillator with a fixed energy and no limit cycle (see Fig. 1). Previous research focusing on a Hamiltonian formulation of oscillations and the emergence of synchrony proved the existence of Kuramoto dynamics in Hamiltonian systems, thus, distinctly linking dissipative to conservative dynamics^51^. While this suggests that chimera patterns might also exist in conservative systems, a proof-of-concept has not been established yet. As we show here, chimera patterns can indeed be observable in certain conservative systems as well as in BECs.

This paper is divided into three parts, each introducing a Hamiltonian systems that can give rise to chimera patterns. The first and most general model is the nonlocal hopping model (NLHM), which can be considered as a generalization of the discrete GPE with nonlocal hopping, or the mean-field theory of the Bose-Hubbard model (BHM)^52–54^ with tunable nonlocal hopping. This model has rarely been studied in the quantum regime ^55,56^. With the introduction of the new characteristic length scale *R* of nonlocal hopping, we show that it can exhibit chimera patterns in the classical regime in both one and two dimensions. We also investigate various properties of these patterns.

In the second part, we introduce a minimal conservative model with local coupling that can give rise to the nonlocal hopping model at an effective level. Nonlocal descriptions are often conveniently used for systems such as gravitational, electric, magnetic, and dipole interactions, even though locality is one of the fundamental principles of physics. These descriptions are accurate when the mediating field is much faster than the dynamics of the particles, allowing the mediating picture to be reduced to an effective particle-particle description with a nonlocal term. Similar effective descriptions can be engineered by adding a mediating channel, such as cavity-mediated global coupling^57^. Additionally, the range of coupling may be tunable in certain systems, such as those with nonlocal diffusive coupling^58^ or long-range coupling mediated by light^59,60^ studied recently. Here, using the same principle, we show that a fast mediating channel can be attached to the existing system, and the adiabatic elimination of the fast channel leads to the NLHM.

In the third part, we aim to identify a conservative physical system that can be accurately described by the NLHM. To this end, we propose a specific physical model based on a two-component BEC in a spin-dependent trap^61^ with coherent oscillations^62,63^. Implementation in BECs, in principle, allows the exploration of both quantum and classical regimes, as well as flexible control of almost all parameters^64,65^. For example, adjusting the particle density and the magnetic field near Feshbach resonances^66^ can change both the rate of particle loss and the strength of nonlinear interactions. In this setup, the hopping originates from the spreading of wavefunctions in the mediating channel governed by a Schrödinger-like equation, so the spatial hopping of atoms is mediated by the matter-wave itself. For this, we employ a mathematical formulation similar to previous studies^55,56^. The loss of ultracold atoms limits the lifetime (which can be critical in certain systems^67^) and can limit the maximum observable range of hopping. We identify an implementable parameter regime in experiments using current technology. Although this regime is not close to the adiabatic limit, we show that chimera patterns still exist. These results suggest that chimera patterns may exist in a wide range of parameter regimes with imperfections, and therefore, may be observable in experiments of ultracold systems using our proposal.

## Results

### Nonlocal hopping model

#### Hamiltonian and dynamic equation

The NLHM model is given by the Hamiltonian:

1 $$\begin{aligned} \mathscr {H} = \mathscr {U}+\mathscr {P} = \frac{U}{2}\sum _{i}|a_{i}|^{4}-P\sum _{i,j}G_{ij}a_{i}^{*}a_{j} \end{aligned}$$

where $a_{i}=\sqrt{n_{i}}e^{i\theta _{i}}$ is a complex number representing the state of site *i*, such that $|a_i|$ is the amplitude, $n_{i}=|a_{i}|^{2}$ is the number of particles or density, and $\theta _{i}$ is the phase. $\mathscr {U}$ is the nonlinear energy with the on-site nonlinear interaction *U*, and $\mathscr {P}$ is the hopping energy with the hopping strength *P*. $G_{ij}$ is the hopping kernel describing the hopping from site $\textbf{r}_{j}$ to $\textbf{r}_{i}$, with $G_{ij}=G_{ji}$. Typically, $G_{ij}$ decreases as the distance $|\textbf{r}_{j}-\textbf{r}_{i}|$ increases and may be characterized by a hopping range *R*. For sufficiently small *R*, the hopping effectively becomes nearest neighbor. In this paper, we use $G_{ij}$ and R derived in Table 1. ﻿This Hamiltonian conserves both the energy and the particle number $N=\sum _{i}n_{i}$. It can also be expressed using the canonical coordinate and momentum $\{q_{i},p_{i}\}$, as well as action and angle variable $\{n_{i},\theta _{i}\}$ (see SM Sect. S1). Note that the hopping term is quadratic $a_{i}^{*}a_{j}$ in the Hamiltonian, which is different from the usual quartic term of a particle-particle interaction $n_{i}n_{j}$ for, say, the Coulomb interaction. Therefore, the corresponding dynamical equation contains the lowest order on-site nonlinearity and the nonlocal linear hopping term:

2 $$\begin{aligned} i\hbar \dot{a}_{i}=U|a_{i}|^{2}a_{i}-P\sum _{j}G_{ij}a_{j} \end{aligned}$$

where $\hbar$ is the Planck constant, which we can set to $\hbar =1$ without loss of generality by rescaling time. Note that this equation is the mean-field equation of the BHM with nonlocal hopping^55,56^. Moreover, the nearest-neighbor variation of this equation is the discrete GPE^68^ and the non-spatial variation is the discrete self-trapping equation^69^.

The dynamic equation of the NLHM can be rewritten in a dimensionless form using the rescaling $a_{i} \rightarrow a_{i}/\sqrt{n_{0}}$, $t \rightarrow (Un_{0}/\hbar )t$, and $P \rightarrow P/(Un_{0})$ where $n_{0}$ is the average number of particles per site. The equation becomes: $i\dot{a}_{i}(t)=|a_{i}|^{2}a_{i}-P\sum _{j}G_{ij}a_{j}$, which depends only on the control parameters of rescaled hopping strength *P* and rescaled hopping radius *R*. Alternatively, Eq. (2) can be written in terms of $\theta _{i}(t)$ and $n_{i}(t)$ as

3a $$\begin{aligned} \dot{\theta }_{i}(t)= & {} Un_{i}-P\sum _{j}G_{ij}\sqrt{\frac{n_{i}}{n_{j}}}\cos (\theta _{j}-\theta _{i}) \end{aligned}$$

3b $$\begin{aligned} \dot{n}_{i}(t)= & {} 2P\sum _{j}G_{ij}\sqrt{n_{i}n_{j}}\sin (\theta _{j}-\theta _{i}) \end{aligned}$$

This explicitly shows that the evolution of the phase $\theta _{i}(t)$ depends on the density $n_{i}(t)$ of the oscillators and vice versa. Even in the very weak hopping regime, they remain coupled to the lowest order. For dissipative systems as illustrated in Fig. 1a, if $\dot{n_i}\sim 0$ after dissipation in the weak coupling regime for all *i*, one can obtain a simplified phase dynamics. This is generally not possible for the conservative case with constant energy since, in general, a large $n_i$ at some site *i* has to be compensated by small $n_j$ at another site or sites to keep the energy constant. This highlights the important role of these conditions for conservative systems in contrast to dissipative systems. The dynamics of the NLHM can be found by solving Eq. (2) using standard numerical methods (see “Methods”), and the results for 1D and 2D are given in the following subsections.

**Table 1:** The *D*-dimensional hopping kernel $G_{D}(r)$ with $r=|\textbf{r}_{j}-\textbf{r}_{i}|$. $K_0$ is the modified Bessel function of the second kind.

	$D=1$	$D=2$	$D=3$
$G_D$	$e^{-r/R}$	$K_{0}(r/R)$	$\frac{1}{r}e^{-r/R}$

#### Chimera patterns in 1D NLHM

**Figure 2:**
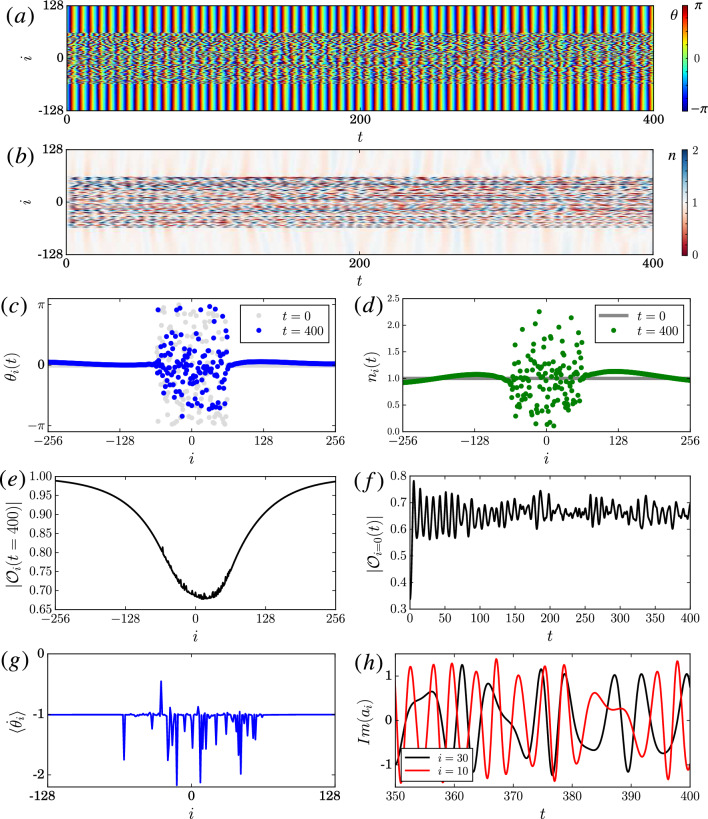
NLHM in 1D with only random initial phases for oscillators. (**a**) Space-time plot of the phase $\theta _i(t)$. (**b**) Space-time plot of the $n_i(t)$. (**c**) Snapshot of $\theta _i(t)$ at $t=0$ and $t=400$. (**d**) Snapshot of $n_i(t)$ at $t=0$ and $t=400$. (**e**) Plot of local order parameter $\mathscr {O}$ at $t=400$. (**f**) Plot of local order parameter $\mathscr {O}$ at $x=0$ over time. (**g**) Average angular frequency $\langle \dot{\theta }_i\rangle$ between $t=0$ to $t=400$. (**h**) The oscillation $Im(a_i)$ of two oscillators near the center. Parameters: $Un_{0}=1$, $P=0.2$, $R=64$, and number of lattice $L=2048$ with no-flux boundary condition and initial density $|a_i|^2=1$. Only the center region is shown for clarity. The hopping kernel $G_{ij}$ is given in Table 1. Dimensionless units and $\hbar =1$ are used.

**Figure 3:**
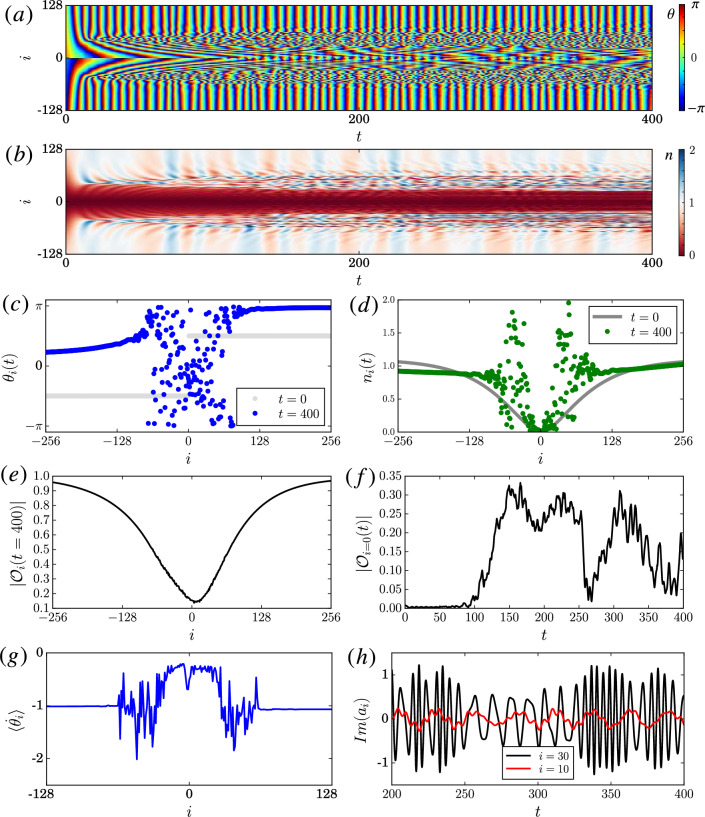
Similar to Fig. 2, but with initial zero density at the center and a phase flip as given in subfigure (**c**) and (**d**). Same parameter as in Fig. 2 with $N = \sum _i|a_i|^2 = L$.

An often used initial condition for chimera patterns is a random phase field^5,24,70,71^, in which chimera patterns can appear after a sufficiently long relaxation time. However, for NLHM, simulations show that the dynamics for such random initial conditions remains incoherent with no clear patterns over time. This is not unexpected since the *spontaneous* emergence of *persistent* patterns in spatially extended systems is typically tied to the notion of an attractor, which does not exist in our conservative model. Instead, incoherent and coherent regions—and, thus, chimera patterns—can sustain themselves over time as shown in Fig. 2a–d starting from initial conditions that are uniform with the exception of random phases (but not amplitudes or densities) in a small region. In particular, the time-averaged angular frequency $\langle \dot{\theta }_{i}\rangle$ as shown in Fig. 2g is uniform in the coherent region and takes on a range of values in the incoherent region, thus, fulfilling the defining property of a chimera state. In terms of the temporal evolution, even though $n_{i}$ is constant initially, the random phases immediately induce fluctuations in the density as shown in Fig. 2b (see animations of the simulations in SM) as expected based on Eq. (3). Such a behavior can not be captured by simplified phase models by construction. To measure the coherence of the phase, we use the local order parameter $\mathscr {O}_i = \sum _j G_{ij} e^{i\theta _j}$^5^. The magnitude $|\mathscr {O}_i| \sim 1$ when all phases $\theta _j$ are the same within the hopping range *R*. As shown in Fig. 2e, $|\mathscr {O}_i|$ takes on a minimum near the center of the incoherent region as expected. Moreover, the local order parameter does not converge but it keeps fluctuating as shown in Fig. 2f (see also Fig. 3f) due to the conservative nature of the system, which prevents relaxation behavior typical for dissipative systems. Figure 2a,c,e,g also indicate that the incoherent region is not fully desynchronized for this initial condition. A much stronger desynchronization can be obtained using an initial condition with both the phase and the amplitude random around the center region, see Fig. S2 in the SM. Hence, the initial amplitude plays a significant role for the characteristics of the observed chimera patterns in conservative systems, while this is typically not the case for dissipative systems, where initial fluctuations in the amplitude tend to be damped away. Another striking observation is that $\langle \dot{\theta }_{i}\rangle$ can behave non-monotonically across the incoherent core (see Figs. 2g and also Fig. 3), whereas it typically changes monotonically with distance from the incoherent core in the dissipative case and in simplified phase models^5^. The incoherent dynamics of the oscillators can be observed in Fig. 2h, where the trajectories of two oscillators inside the incoherent region are shown. The specific value of the hopping strenght $P>0$ does not affect the chimera patterns qualitatively. However, for uniform initial conditions in the amplitude, the fluctuations in the amplitude can decrease when *P* decreases as shown in Fig. S3 in SM.

While this could suggest that a simple phase description is sufficient in some special cases, such a simplification is generally not possible as already discussed above. Specifically, one distinctive feature of the NLHM is that the local phase oscillators can oscillate at any amplitude because of the lack of a limit cycle attractor. This can be observed using an initial condition with different amplitudes. An example is given in Fig. 3c,d where the initial density drops to zero and the phase changes by $\pi$ at the center (this is a cross-section of a vortex phase initial condition, see next section for more details). As suggested by previous studies^27,28^, interesting chimera patterns can be formed spontaneously from such a regular initial condition. Here, the local phase incoherence and local density fluctuation around the center increase over time as shown in Fig. 3a,b. As Fig. 3h shows, the instantaneous frequency of the oscillators near the center can also change significantly over time. In particular, these oscillations have near zero amplitude as shown in Fig. 3b,h. In contrast, for the corresponding chimera patterns formed in dissipative systems with self-sustained oscillators the oscillations typically evolve close to the limit cycles in the weak coupling regime^6^.

#### Chimera patterns in 2D NLHM

**Figure 4:**
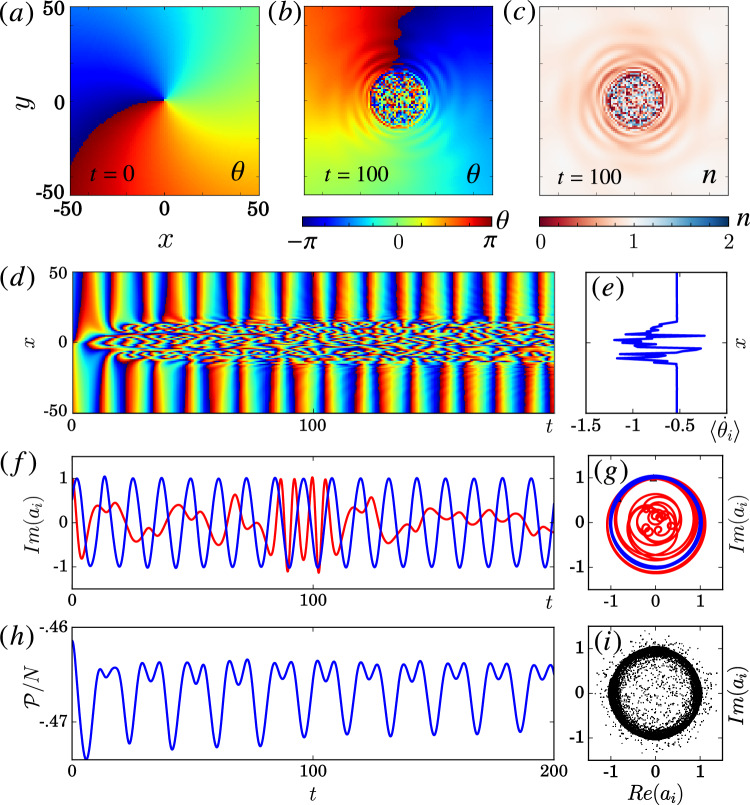
Chimera patterns in the 2D NLHM given by Eq. (1). (**a**) The initial phase with uniform amplitude $|a_i|=1$ at time $t=0$. (**b**,**c**) Phase $\theta _i$ and number of particle $n_i=|a_i|^{2}$ at $t=100$. (**d**) Time evolution of the phase $\theta _i(t)$ for the cross-section $y=0$. (**e**) Averaged local rotation speed $\langle \dot{\theta }_{i}\rangle$ over the time interval in (**d**). (**f**) Time evolution of the points near the center $(x,y)=(-5,0)$ (red) and far away $(-100,0)$ (blue). (**g**) Local phase space trajectory of (**f**). (**h**) Hopping energy per particle $\mathscr {P}/\mathscr {N}$ variation over time. (**i**) Phase portrait of all points at $t=100$. Parameters: $Un_{0}=1$, $P=0.5$, $R=16$, and length $L=256$ with no-flux boundary condition. Only the core region is shown for clarity. The hopping kernel $G_{ij}$ is given in Table 1. Dimensionless units and $\hbar =1$ are used.

**Figure 5:**
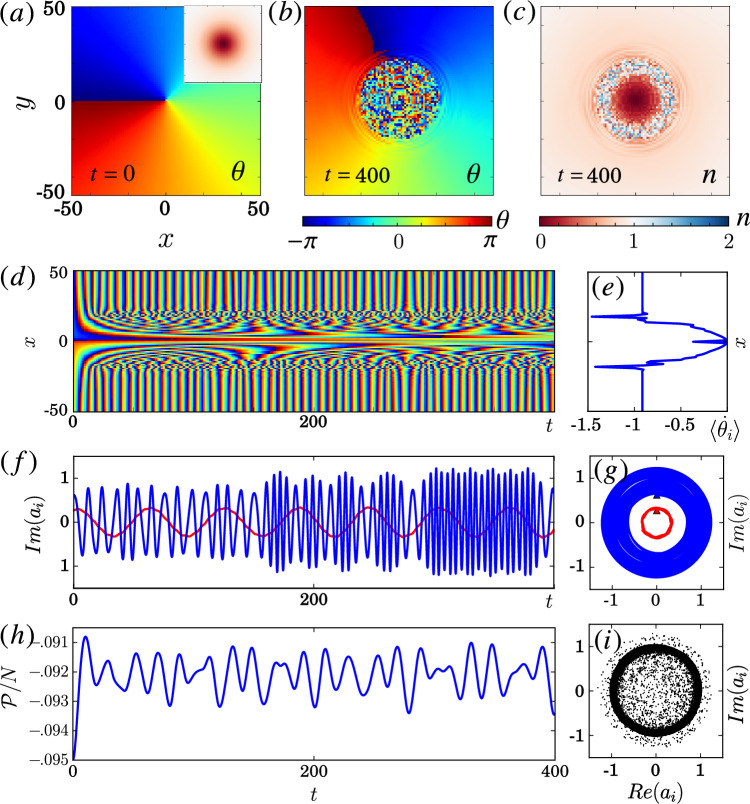
Similar to Fig. 4 but with vortex initial condition given by the phase in (**a**) and the density in the inset, and with a weaker hopping strength. The points are $(x,y)=(-5,0)$ (red) and $(-15,0)$ (blue) in (**f**). For (**g**) and (**i**), $t=100$. Parameters: $Un_{0}=1$, $P=0.1$, $R=16$, and size $L=256$ with no-flux boundary condition.

Similarly to the 1D system, an initial condition with random phase regions can sustain itself over time in 2D. Here, we focus on such chimera patterns, in particular those where an incoherent region forms spontaneously around a phase singularity^25,27,28^. These patterns benefit from a topological protection in the sense that the incoherent core is robust against fluctuations in the phases. The first initial condition we examine is a spiral phase initial condition that is locally phase coherent everywhere except the center, with uniform density, as illustrated in Fig. 4a (see also “Methods”). With this initial condition, the system can spontaneously evolve into a state with a small incoherent core surrounded by a large spatially coherent region as shown in Fig. 4b for the phase field. Moreover, the density is randomized near the same core region in Fig. 4c. As shown by the dynamics of a cross-section in Fig. 4d, this spatial structure is sustained over long times (see Fig. S4 for snapshots and animations in SM). In addition, the same patterns can be observed even when the system size *L* and also *R* are increased (see Fig. S5 in SM). The local dynamics of the two oscillators in Figs. 4f,g clearly shows the difference between two regions: $a_{i}$ oscillates regularly far from the core, but not close to it. As in the 1D system, the incoherent region can only appear if the hopping range *R* is sufficiently large, here $R \gtrsim 3$. Moreover, with nearest-neighbor hopping, the system reduces to the discrete GPE so that the incoherent region spreads out and interferes like a wave (see Fig. S8 in SM). All of these features are consistent with previous observations of chimera cores for driven-dissipative systems with self-sustained oscillators^24,25,28^. The distinct features in 2D are similar to the ones in 1D. This is shown in Fig. 4e–g for the angular frequencies and the trajectories in phase space. Note especially the strong variations in the average local rotation speed. In particular, the oscillators can exhibit significant variations in amplitudes as follows from Fig. 4c,g and the phase portrait in Fig. 4i that shows the phase and amplitude of all oscillators at a given moment in time. We would like to point out that after the formation of the chimera core, the pattern persists over the longest time scales we were able to simulate ($>1000$ spiral rotations). This observation suggests that if a random phase core is used as an initial condition, the chimera core pattern also persists over such long times scale. This is indeed what we observe (see Fig. S6 in SM).

The important amplitude-dependent dynamics without limit cycles can be clearly observed for the vortex phase initial condition with amplitude going to zero at center in Fig. 5 (see Fig. S7 for snapshots in SM), with a weaker hopping $P=0.1$. Similar to the 1D case discussed above, the fluctuations in the amplitude remain close to the initial condition for small *P*. In particular, oscillators with different amplitudes have different oscillating frequencies even in the weak hopping regime according to Eq. (3) with small corrections arising from the weak hopping. More importantly, as a conservative Hamiltonian system, it has time reversal symmetry and it conserves both quantities $\mathscr {H}$ and *N* (see Fig. S9 and animations in SM). This leads to persistent fluctuations or ripples as observed in Fig. 4b–d, which would be damped away in a dissipative system quickly. In addition, the results of the backward time evolution of the core region are very delicate. With a small perturbation, the background can evolve back to nearly the same state at $t=0$, but the core remains incoherent (see Fig. S9 in SM), which again signifies the difference between the two regions (see Sect. S4 and animations in SM). This suggests that the Poincaré recurrence time to a regular spiral—the time it takes to return within an arbitrarily small but finite distance to the original state (modulo possible rotations or translations)—is large and that the probability to encounter a regular spiral is zero in the infinite system size limit.

Moreover, the hopping energy $\mathscr {P}$ is not constant as shown in Fig. 4h even though the total energy $\mathscr {H}$ is constant. Hence, there is a conversion between $\mathscr {P}$ and $\mathscr {U}$ over time. This is different from a simple coherent and uniform distribution $a_i=\sqrt{n_0}$ having an energy per particle given by $\mathscr {H}/N = Un_0^2/2 - Pn_0$ with constant $\mathscr {P}$ and $\mathscr {U}$. Note that all chimera patterns considered here do not correspond to ground states of the Hamiltonian but are excited states.

In realistic experimental systems, a small amount of particle loss typically exists and can be modeled phenomenonlogically by the term $U\rightarrow U-iU_{loss}$. Intuitively, the dynamics should not change significantly if the loss of the particles is less than half of the initial number of particles given by the condition $U_{loss} t/\hbar \lesssim 1$, Indeed, chimera patterns can, for example, still be observed with $U_{loss}/U=0.02$ at a sufficiently short time (Fig. S11 in SM). Further details about such loss in 1D (Fig. S10 in SM) and 2D (Fig. S11 in SM) are discussed in Sect. S4 in SM.

### Mechanism for nonlocal hopping and the minimal model

#### Mediating mechanism

**Figure 6:**
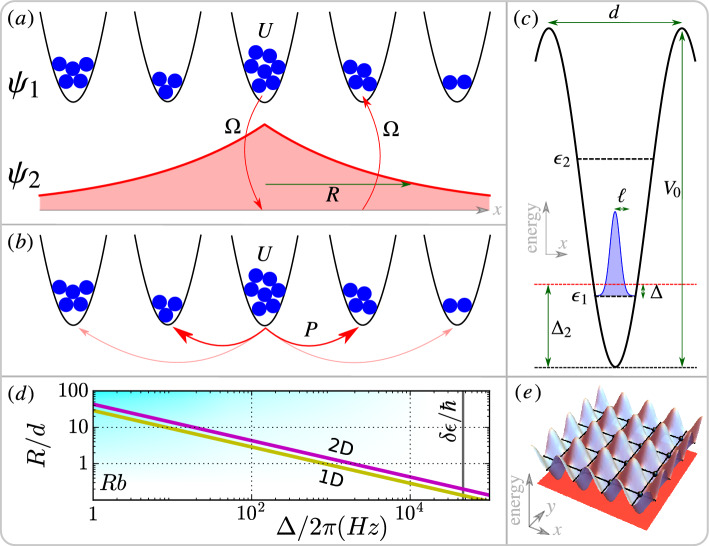
Illustration of mediated hopping. (**a**) Two-component model: Particles with on-site interaction *U* are trapped (denoted by $\psi _1$) but can be converted into a mediating state (denoted by $\psi _2$) that can propagate freely. It is eventually converted back to nearby sites, giving rise to a characteristic hopping range *R*. (**b**) Effective model with hopping strength *P* after adiabatically eliminating the fast mediating channel. (**c**) Periodic lattice with spacing *d* and lattice depth $V_{0}$: Trapped bosonic particles can be described by local ground state wavefunctions with width $\ell$ and energy $\epsilon _{1}$ (with energy gap $\delta \epsilon = \epsilon _{2}-\epsilon _{1}$). *P* and *R* can be controlled by the coherent oscillation frequency $\Omega$ and the detuning $\Delta =\Delta _{2}-\epsilon _{1}/\hbar$ between localized states and mediating states. (**d**) *R* can be adjusted by $\Delta$, see text for details. (**e**) 2D periodic lattice considered in Fig. 8.

The key idea for the mediating mechanism is to attach an inter-convertible mediating channel (labelled by $\psi _2$) to trapped states (labelled by $\psi _1$) as illustrated in Fig. 6a. With direct hopping, increasing the energy barrier between neighboring sites decreases both the hopping strength and the hopping range together. In contrast, if the particles can be converted into fast mediating states that do not experience any energy barrier, then the particles can physically jump much further away. Mathematically, this channel can be eliminated adiabatically (as done, for example, in^5,72^ for non-Hamiltonian systems), resulting in an effective nonlocal model (see Fig. 6b) with independently adjustable on-site nonlinearity, hopping strength, and hopping range that can be tuned from nearest-neighbor to global hopping.

#### Minimal model

A minimal mathematical model that captures the concepts of the mediating channel discussed above takes the form:

4a $$\begin{aligned} i\hbar \dot{\psi }_{1}(\textbf{r},t)= & {} U|\psi _{1}|^{2}\psi _{1}+\hbar \Omega \psi _{2} \end{aligned}$$

4b $$\begin{aligned} i\hbar \dot{\psi }_{2}(\textbf{r},t)= & {} -\hbar \kappa \nabla ^{2}\psi _{2}+\hbar \Omega \psi _{1}+\hbar \Delta \psi _{2} \end{aligned}$$

for the localized $\psi _1$ and mediating $\psi _2$ components respectively. The corresponding Hamiltonian is given in Eq. (6) with appropriate parameters. The inter-conversion is governed by a detuning $\Delta$, which conserves the number of particles, and a coherent coupling with coherent oscillation frequency $\Omega$. This coupling may alternatively be referred to as Rabi coupling or Josephson coupling, depending on the physical systems being studied^62,63,73^. Eq. (4b) is essentially the Schrödinger equation for free particles with inverse mass $\kappa =\hbar /(2m)>0$ and so the particles can propagate outward. The additional detuning in the far-detuned regime $|\Delta | \gg |\Omega |$ can ensure the mediating idea is well-defined: The number of particles $N_{j}=\int d\textbf{r}|\psi _{j}|^{2}$ in the mediating channel $N_{2}\ll N_{1}\approx N$ can be neglected. Note that this model is not captured by the framework of nonlocal diffusive coupling^58^. It is explicitly constructed to always preserve the conservation properties of the underlying Hamiltonian system, even when adiabatic elimination is applied.

#### Adiabatic elimination

**Figure 7:**
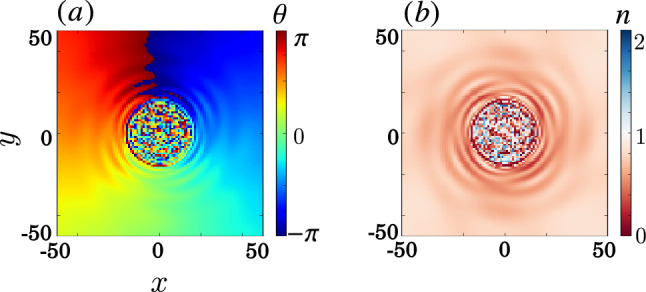
Chimera patterns in the minimal model with the direct simulation using Eq. (4) at $t=100$ similar to Fig. 4b,c. The setting is the same as in Fig. 4 but with parameters $\Delta =16$, $\Omega =\sqrt{8}$, $U=1$, and $\kappa =4096$.

Suppose $\psi _{1}$ evolves much slower than $\psi _{2}$, then we can apply adiabatic elimination by setting $\dot{\psi }_{2}=0$^74^. The solution of $-\kappa \nabla ^{2}\psi _{2}+\Omega \psi _{1}+\Delta \psi _{2}=0$ in the unbounded isotropic space with translation invariant is given by the convolution $\psi _{2}(\textbf{r},t)=-(\Omega /\Delta )G_{D}(\textbf{r})*\psi _{1}(\textbf{r},t)$, where $G_{D}(\textbf{r})$ is the *D*-dimensional hopping kernel, or Green's function, as listed in Table 1, with hopping radius $R=\sqrt{\kappa /|\Delta |}$. Note that $\Delta >0$ is required for the solution of confined hopping kernels (see the form of $\psi _2$ in Fig. 6a), while $\Delta <0$ leads to wave-like solution. Substituting this solution back into Eq. (4a), we can get the continuum NLHM:

5 $$\begin{aligned} i\hbar \dot{\psi }(\textbf{r},t) = U|\psi |^2\psi - P\int d\textbf{r}'G(\textbf{r},\textbf{r}')\psi (\textbf{r}',t), \end{aligned}$$

where the summation is replaced by an integral with hopping strength $P=\hbar \Omega ^{2}/\Delta$. As shown in Fig. 7, the continuous NLHM well-approximates the discrete NLHM results from Fig. 4.

### Mediated hopping in ultracold atomic systems

#### Hamiltonian and dynamic equation

An ultracold atomic system of a general two-component GPE in a spin-dependent trap with coherent conversion is given by the Hamiltonian:

6 $$\begin{aligned} \mathscr {H}=\sum _{i=1,2}\left( \mathscr {H}_{i}+\frac{1}{2}\mathscr {U}{}_{ii}\right) +\mathscr {U}{}_{12}+\mathscr {R}, \end{aligned}$$

with

7 $$\begin{aligned} \mathscr {H}_{i}= & {} \int d\textbf{r}\left( \frac{\hbar ^{2}}{2m_i}|\nabla \psi _{i}(\textbf{r})|^{2}+V_{i}(\textbf{r})|\psi _{i}(\textbf{r})|^{2}\right) , \end{aligned}$$

8 $$\begin{aligned} \mathscr {U}_{ij}= & {} g_{ij}\int d\textbf{r}|\psi _{i}(\textbf{r})|^{2}|\psi _{j}(\textbf{r})|^{2}, \end{aligned}$$

9 $$\begin{aligned} \mathscr {R}= & {} \sum _{i=1,2}\hbar \Delta _{i}\int d\textbf{r}|\psi _{i}(\textbf{r})|^{2}+\hbar \Omega \int d\textbf{r}\left( \psi _{1}^{*}(\textbf{r})\psi _{2}(\textbf{r})+\psi _{2}^{*}(\textbf{r})\psi _{1}(\textbf{r})\right) , \end{aligned}$$

and with the normalization $N=N_{1}+N_{2}$ where $N_{i}=\int d\textbf{r}|\psi _{i}(\textbf{r})|^{2}$ is the number of particles for each component. $m_i$ is the mass of the particles, $V_{i}(\textbf{r})$ is the trap potential, $g_{ij}$ is the two-particle collision coefficient, and we assume $g_{12}=g_{22}=0$ for the moment (see explanation below for non-zero case). The coherent oscillation term ${{\mathscr {R}}}$ represents the inter-conversion between the two components with the spatially homogeneous coherent oscillation frequency $\Omega$ and the detuning $\Delta _{i}$. By setting $V_i=0$, $m_1 \rightarrow \infty$, and $\Delta _{1}=0$, we arrive at the Hamiltonian for the minimal model discussed above. When a small nonlinearity exists in the mediating channel, the effective detuning becomes $\Delta \rightarrow \Delta +g_{12}|\psi _1|^2+g_{22}|\psi _2|^2$ if $\psi _i$ is uniform. Hence, the hopping radius decreases for $g_{ij}>0$ which is typical for atomic systems. Note that when $|\psi _i|^2$ is small, the nonlinear effect can be ignored. It can be achieved by decreasing the density, which is one of the main technique used in the analysis of real systems below.

Mathematically, Eq. (4) can be obtained by setting appropriate parameters for the system described by Eq. (6). In particular, the absence of kinetic energy term in Eq. (4a) requires $m_1 \rightarrow \infty$. However, the mass *m* of inter-convertible atomic systems are the same, so $m_i = m$. To circumvent this, we can increase the effective mass; for example, by placing the atoms in a periodic lattice. This can be achieved by additionally setting $V_2=0$, $V_{1}$ to be periodic, and $\Delta _1=0$. Then the dynamic equation becomes^75^:

10a $$\begin{aligned} i\hbar \dot{\psi }_{1}(\textbf{r},t)= & {} \left( -\hbar \kappa \nabla ^{2}+V_{1}+g_{11}|\psi _{1}|^{2}\right) \psi _{1}+\hbar \Omega \psi _{2} \end{aligned}$$

10b $$\begin{aligned} i\hbar \dot{\psi }_{2}(\textbf{r},t)= & {} \left( -\hbar \kappa \nabla ^{2}+\hbar \Delta _{2}\right) \psi _{2}+\hbar \Omega \psi _{1} \end{aligned}$$

Only the positive detuning $\Delta = \Delta _{2} - \epsilon _{1}/\hbar >0$ is considered here as illustrated in Fig. 6c.

#### Mapping to effective NLHM

Note that direct adiabatic elimination does not work if states with high energy $\epsilon _{i>1}$ are occupied. This is because high energy states do not evolve slowly compared to the mediating component. To avoid occupying higher energy levels, we can confine the system to local ground states $\phi ({\textbf {r}})$ with energy $\epsilon _{1}$ and prevent excitation by choosing a suitable detuning such that $\epsilon _{2}-\epsilon _{1} \gg \hbar \Delta \gg \hbar |\Omega |$ (see Fig. 6c). Under these constraints, along with adiabatic elimination, we can show (Sect. S2 in SM) that Eqs. (10a) and (10b) reduce to the exact form of Eq. (2) with $U=g_{11}\int |\phi |^4$, $P=\hbar \Omega ^{2}/\Delta$, hopping kernel $G_{D}(r)$ in Table 1, and

11 $$\begin{aligned} R=C_{D}\left( \frac{d}{2\ell }\right) ^{\frac{D}{2}}\sqrt{\frac{\kappa }{\Delta }} \end{aligned}$$

for $d\gg 2\ell$, where $C_{D}$ is a constant. Intuitively, particles staying in the mediating channel for a longer time have a larger hopping range $R\sim \Delta ^{-1/2}$. Since the effective conversion region has a characteristic length scale $2\ell$ in a unit lattice with length *d*, scaling with $2\ell /d$ is expected. Indeed, we have the effective scaling $\Delta \rightarrow \Delta _{eff}=(2\ell /d)^{D}\Delta$. The self-consistency condition for adiabatic elimination is $\hbar \Delta \gg Un_{0},P$ assuming all $n_{i}\sim n_{0}$ ($n_{0}$ is the average number of particles per site). In this effective NLHM, $a_{i}$ in Eq. (1) represents the state of a localized wavepacket at site *i*. Moreover, the kernel $G_{ij}$ in Eq. (1) describes the matter-wave mediated hopping with wavepackets annihilated at site *j* and created at site *i*.

#### Optical lattice

The system discussed above requires a particle that is inter-convertible, which can be an atom with two different hyperfine states. A candidate is the Rubidium atom with hyperfine states $|F=1, m_F=-1\rangle$ and $|F=1, m_F=0\rangle$ which has been realized in a spin-dependent trap^61^. Suppose the trapping potential is sinusoidal $V_{1}({\textbf {r}})=V_{0}\sum _{\sigma }\sin ^{2}(kx_{\sigma })$ with wavelength $\lambda$, wavenumber $k=2\pi /\lambda$, lattice spacing $d=\lambda /2$, and trap depth $V_{0}$. The summation is taken over the lattice trap dimension as shown in Fig. 6c or e. For sufficiently large $V_{0}$, all direct hopping can be suppressed, and the local ground states at trap minima can be approximated by a Gaussian $\phi _{\sigma }(x_{\sigma })=e^{-\pi x^{2}/(2\ell _{\sigma }^{2})}/\sqrt{\ell _{\sigma }}$ with $\ell _{\sigma }=\sqrt{\pi \hbar /(m\omega _{\sigma })}$. In this setting, the nonlinearity is enhanced by the high density since $U=g_{11}/W$ with effective volume $W=2^{3/2}\ell _{x}\ell _{y}\ell _{z}$. The constant can be found by numerical fitting, which gives $C_{D}\approx 1$ (see Sect. S3 and Fig. S1 in SM).

#### Achievable hopping range

For the hopping to be considered nonlocal, $R>d$ must be satisfied. An example of Rubidium atoms is shown in Fig. 6d with $d=395$ nm and a deep trap $s=40$ (expressing $V_{0}=sE_{R}$ in recoil energy $E_{R}=\hbar \kappa k^{2}$). With such a large *s*, as studied before^52^, the overlap between wavefuncion of neighboring cell is very small, the direct hopping is weak, and the system becomes a Mott insulator in the quantum regime. Nevertheless, mediated hopping can completely replace the direct hopping (with order $R\sim d$, see Fig. 6d) and allow real time control. Since $\Omega$, $\Delta$, and *U* can be easily adjusted in experiments, there seems to be no upper bound on *R*. From a practical point of view, however, it is limited by the lifetime $\tau$ and experimental duration. A simple estimation of $\tau \sim 1$s gives a maximum $R\sim 30d$ as shown in Fig. 6d.

#### Tuning nonlinearity and loss

The regime with competitive $P\sim Un_{0}$ is the most interesting. However, a BEC in a 3D optical lattice using the parameters given above has a strong nonlinearity $U/\hbar =2\pi \times 2.23$ kHz, which demands a large $\Delta$ and, consequently, a small *R*. *U* can be reduced by the use of two tuning techniques: Decreasing the density, or utilizing the Feshbach resonance. The latter method can experimentally tune the nonlinearity over many orders of magnitude^66^. The former method is preferable because both nonlinearity and collision loss can be decreased simultaneously. In 1D and 2D lattices, the non-lattice dimension can be weakly trapped to reduce the density, resulting in a lattice of disk and cigarette-shaped wavefunctions respectively^76,77^. In this case, the dominant loss is the two-particle loss in the localized component. The rate of the two-particle loss can be estimated by $U_{loss}=\hbar L_{11}/W$ and therefore half-life $\tau =W/L_{11}$ with two-particle loss rate $L_{11}$^67^. This implies that $\tau \sim \ell _{z}$ in 2D, so increasing $\ell _{z}$ can improve the BEC lifetime.

#### Chimera patterns in BECs

**Figure 8:**
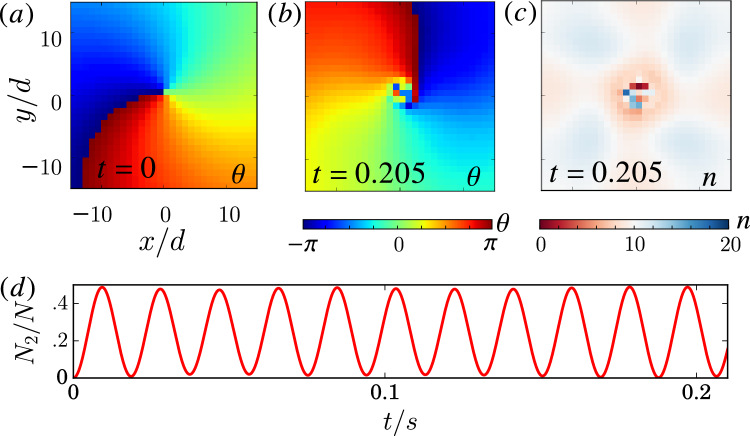
Chimera patterns in BECs. (**a**) Initial phase $\theta _i$ with a uniform number of particles per lattice site $n_i=10$. (**b**,**c**) $\theta _i$ and $n_i$ of the state at time $t=205$ ms. The simulation is based on Eq. (10) in the 2D lattice given by Fig. 6e with $100d\times 100d$ and the no-flux boundary condition. We spatially average over the lattice units. (**d**) Inter-conversion between two components. For the optical lattice, we use Rubidium $^{87}$Rb with $s=40$ and $d=395\text{ nm }$ which gives and $\ell _{x}=\ell _{y}=0.22d$. Additional parameters: $\Delta =2\pi \times 48\text{ Hz }$, $\Omega =2\pi \times 32\text{ Hz }$. The density is decreased by using $\ell _{z}=200\ell _{x}$, and the nonlinearity is weaken 10 times by using Feshbach resonance. The estimated values are $Un_{0}/\hbar \approx 2\pi \times 19\text{ Hz }$, $P\approx 2\pi \times 16\text{ Hz }$, $R\approx 6d$, and $\tau \approx 5\text{ s }$.

The derivation of effective models implies that chimera patterns can also be observed in certain parameter regimes for Eqs. (4) and (10). The question is: can such parameter regimes be achieved in BEC experiments with current technology? The possible existence of chimera patterns in ultracold atoms is established in a parameter regime given in Fig. 8, based on a full simulation of Eqs. (10). Similar to Fig. 4, a random core appears eventually. Figure 8d shows the coherent oscillation between the two components with frequency $\sim \sqrt{\Omega ^2+\Delta ^2}$. Note that most of the atoms can be converted back after a full period, which confirms the physical picture discussed in Fig. 6a and is consistent with previous works^55,56^. The regime $|\Delta |\gtrsim |\Omega |$ studied here is not in the far-detuned regime and may not be well described by NLHM, yet chimera patterns can still be observed in simulations. This suggested that chimera patterns do exist in a wide range of parameter regimes. As experimental techniques continue to improve, it will be possible to explore the adiabatic regime more closely.

Experimentally, the initial state can be prepared starting from a uniform BEC. Thousands of optical lattice sites^77–79^ can be created with $V_{1}$ adiabatically turned on until the direct hopping is suppressed and mediated hopping begins to dominate. The energy shift induced by a short light-pulse can then be used to create any desired initial phase. The system states and dynamics may be detected by using various techniques such as optical readout, time of flight techniques, or matter-wave interference^80,81^. The loss $U_{loss}/U \approx 0.017$ here is comparable with the discussion in the minimal model. Note that a small amount of loss can cause the BEC system to follow the classical trajectory^82^, and so each site can be well-described by a classical mean-field amplitude and phase. At the same time, our simulations suggest that chimera core patterns in 2D are particularly robust due to their topological structure. Specifically, if we start with a chimera core initial condition it can persist over long times. This is particularly useful if the lifetime of BECs is further limited in a given experiment by other experimental imperfections. All of this suggests that chimera patterns should be observable in experimental BECs.

## Discussion

In summary, our work presents a Hamiltonian formulation of chimera states and demonstrates the existence of chimera patterns in three conservative Hamiltonian systems. The NLHM used in our study is a direct analogue of the nonlocal CGLE^5^, and our appoarch allows for the application of existing techniques in ultracold atoms and can be readily generalized to the quantum regime. Our simulations in realistic parameter regimes of BECs suggest that chimera patterns should be observable in experiments with ultracold systems using current technology. Additionally, our results suggest that the persistence of the incoherent region and the formation of a chimera core starting from a vortex or spiral initial condition in 2D are two distinct indicators of correct implementation of mediated nonlocal hopping, as opposed to local hopping which would result in the smoothing out of the incoherent region over time.

Our results in this paper are based on classical conservative Hamiltonian systems, which provides a new avenue to understand chimera patterns. These results may be extended into the quantum regime, since all of the physical processes that we analyzed are coherent and conserve both energy and particles. Equations (1)–(2), (4)–(10) can be quantized, and Eq. (1) becomes the Bose-Hubbard model with tunable mediated hopping^55^. This opens the door for the exploration of exotic condensed-matter states, such as supersolid states and quantum vortices with topological defect, in addition to other long-range effects^54,83^. The technique that we presented suggests that experimental studies of the synchronization and chimera patterns of a large number of oscillators may be feasible in quantum systems^37,84–88^. We hope that our work here will motivate further studies of chimera patterns on ultracold atoms and quantum systems.

## Methods

The numerical methods we used for solving Gross-Pitaevskii equations in Fig. 8 are the fourth-order time splitting method^89^. This method for the conservative systems automatically conserve the particle number. For all the other results, we used the standard fourth-order Runge-Kutta. The geometry used in the simulations is a square lattice with size *L* and the no-flux boundary condition. For the spiral initial condition, uniform density $|a_{i}|=\sqrt{n_{0}}$ is used and the state is given by $a_{i}(t=0)=\sqrt{n_{0}}e^{i(k_{s}r-\tan ^{-1}(y/x))}$ with $r=\sqrt{(x^{2}+y^{2})}$. For the vortex-like initial condition, the state is given by $a_i(t=0) = A_i e^{i \tan ^{-1}(y/x)}$ with $A_i = 1-e^{-r/R_{vortex}}$ and $R_{vortex}$ is the length scale of the vortex. We use $R_{vortex}=R$ in this manuscript. For the system with a mediating channel, the channel is initially empty $\psi _2=0$.

## Supplementary Information

Supplementary Information 1.

Supplementary Information 2.

Supplementary Information 3.

Supplementary Information 4.

Supplementary Information 5.

Supplementary Information 6.

## Data Availability

The datasets generated during the current study are available from the corresponding author on reasonable request.
