# Supplementary material for: Chimera patterns in conservative Hamiltonian systems and Bose–Einstein condensates of ultracold atoms
Source: Sci Rep. 2023 May 26;13:8590. doi: 10.1038/s41598-023-35061-3 (PMC10220079; doi:10.1038/s41598-023-35061-3)
Supplement: Supplementary file 6 — Supplementary Information 6. [file 41598_2023_35061_MOESM6_ESM.pdf]

# Supplementary Materials: Chimera patterns in conservative systems and ultracold atoms with mediated nonlocal hopping

## I. ALTERNATIVE HAMILTONIANS OF THE NONLOCAL HOPPING MODEL

The Hamiltonian of the NLHM is given by

$$\mathcal{H} = \frac{U}{2}\sum_i |a_i|^4 - P\sum_{i,j} G_{ji}a_i^* a_j, \tag{S1}$$

with $G_{ij} = G_{ji}$ and $G_{ii} = 0$. This Hamiltonian can be represented in a few different canonical variables (see [S1, S2] for example). Suppose the canonical coordinate and momentum variables are $q_i$ and $p_i$ respectively, then we can define

$$a_i = \frac{1}{\sqrt{2}}(q_i + ip_i), \tag{S2}$$

$$a_i^* = \frac{1}{\sqrt{2}}(q_i - ip_i). \tag{S3}$$

With this transformation, the Hamiltonian becomes

$$\mathcal{H} = \frac{U}{8}\sum_i \left(q_i^2 + p_i^2\right)^2 - \frac{1}{2}P\sum_{i,j} G_{i,j}(q_i q_j + p_i p_j), \tag{S4}$$

Similarly, we can define the action and angle to be $n_i$ and $\theta_i$ respectively, such that $a_i = \sqrt{n_i}e^{i\theta_i}$, or

$$n_i = \frac{1}{2}\left(q_i^2 + p_i^2\right), \tag{S5}$$

$$\theta_i = \tan^{-1}\left(p_i/q_i\right). \tag{S6}$$

Now, the Hamiltonian becomes

$$\mathcal{H} = \frac{U}{2}\sum_i n_i^2 - P\sum_{i,j} G_{i,j}\sqrt{n_i n_j}\cos(\theta_j - \theta_i). \tag{S7}$$

Note that $n_i$ may be interpreted as the (mean-field) number of particle at site $i$. Hence, the conservation of the total number of particles implies the quantities $\sum_i |a_i|^2$, $\sum_i \left(q_i^2 + p_i^2\right)$, and $\sum_i n_i$ are constant. Moreover, the Hamiltonian is invariant under the transformation $a_i \to a_i e^{i\theta_0}$ with arbitrary global phase.

In the continuum limit, such as the adiabatic elimination of the simplest two-component model in the the main text, the corresponding Hamiltonian can be obtained by replacing $a_i \to \psi(\mathbf{r})$, $\sum_i \to \int d\mathbf{r}$, $\sum_{i,j} \to \int\int d\mathbf{r}d\mathbf{r}'$ and $G_{i,j} \to G(\mathbf{r},\mathbf{r}')$. Explicitly, the Hamiltonians are:

$$\mathcal{H} = \frac{U}{2}\int d\mathbf{r}|\psi(\mathbf{r})|^4 - P\int\int d\mathbf{r}d\mathbf{r}'G(\mathbf{r},\mathbf{r}')\psi^*(\mathbf{r})\psi(\mathbf{r}'), \tag{S8}$$

$$\mathcal{H} = \frac{U}{8}\int d\mathbf{r}\left(q(\mathbf{r})^2 + p(\mathbf{r})^2\right)^2 - \frac{1}{2}P\int\int d\mathbf{r}d\mathbf{r}'G(\mathbf{r},\mathbf{r}')(q(\mathbf{r})q(\mathbf{r}') + p(\mathbf{r})p(\mathbf{r}')), \tag{S9}$$

$$\mathcal{H} = \frac{U}{2}\int d\mathbf{r}\left(n(\mathbf{r})\right)^2 - P\int\int d\mathbf{r}d\mathbf{r}'G(\mathbf{r},\mathbf{r}')\sqrt{n(\mathbf{r})n(\mathbf{r}')}\cos(\theta(\mathbf{r}') - \theta(\mathbf{r})). \tag{S10}$$

## II. HOPPING IN ULTRACOLD ATOMS WITH A PERIODIC LATTICE

We start from the equations in the main paper:

$$i\hbar\dot{\psi}_1(\mathbf{r},t) = \left(-\hbar\kappa\nabla^2 + V_1 + g_{11}|\psi_1|^2\right)\psi_1 + \hbar\Omega\psi_2, \tag{S11}$$

$$i\hbar\dot{\psi}_2(\mathbf{r},t) = \left(-\hbar\kappa\nabla^2 + \hbar\Delta_2\right)\psi_2 + \hbar\Omega\psi_1. \tag{S12}$$

In general, adiabatic elimination works best when the first component evolves the slowest [S3]. However, no such choice exist for an arbitrary wavefunction of Eq. (S11), but it exists when the dynamics are confined to the lowest energy band since the excitations have fast dynamics. We derive the effective model with these two assumptions.

Note that there is no basis that is simultaneously good for both equations; although, the good basis for the localized and mediating equation are the Wannier basis and Fourier basis respectively. For the system here, it is easier to understand in the Wannier basis $\{w_{mn}(\mathbf{r})\}$ [S4, S5] for a periodic lattice, where $n$ is the energy band index and $m$ is the lattice site index. In this new basis, the wavefunctions can be represented by $\psi_1(\mathbf{r},t) = \sum_{mn} a_{mn}(t) w_{mn}(\mathbf{r})$ and $\psi_2(\mathbf{r},t) = \sum_{mn} b_{mn}(t) w_{mn}(\mathbf{r})$ respectively. Substituting the transformation back into Eq. (S11) and (S12), we have

$$i\hbar \dot{a}_{mn}(t) = \epsilon_{mn} a_{mn} + U|a_{mn}|^2 a_{mn} + \hbar\Omega b_{mn}, \qquad \text{for } n = 1, \tag{S13}$$

$$i\hbar \dot{b}_{mn}(t) = \hbar \sum_{kl} c_{mnkl} b_{kl} + \hbar\Delta_2 b_{mn} + \hbar\Omega a_{mn}, \tag{S14}$$

where

$$\epsilon_{mn} = \int_V d\mathbf{r} \left(\hbar\kappa|\nabla w_{mn}|^2 + V_1|w_{mn}|^2\right), \tag{S15}$$

$$U = g_{11} \int_V d\mathbf{r}|w_{mn}|^4, \tag{S16}$$

$$c_{mnkl} = \kappa \int_V d\mathbf{r} \nabla w^*_{mn}(\mathbf{r}) \nabla w_{kl}(\mathbf{r}), \tag{S17}$$

Note that we assume $a_{mn} = 0$ for all $n > 1$. We also assume that the trap potential $V_1$ is sufficiently deep so that there is no direct hopping. In this setting, the eigenenergy $\epsilon_{m1} = \epsilon_0$ is a constant. Hence, we can shift the energy $\Delta_2 \to \Delta := \Delta_2 - \epsilon_0/\hbar$ using the transformation $a_{mn} \to a_{mn} e^{-i\epsilon_0 t}$. If the energy gap is large $\epsilon_{m2} - \epsilon_{m1} \gg \hbar\Delta$, then we can ignore the resonance with the higher band index $n > 1$. Furthermore, with initially empty excited states, i.e. $a_{mn}(t = 0) = 0$ for $n > 1$, no excited states will be populated because there are no resonance with those states. Written explicitly:

$$i\hbar \dot{a}_{m1}(t) = U|a_{m1}|^2 a_{m1} + \hbar\Omega b_{m1}, \tag{S18}$$

$$i\hbar \dot{b}_{m1}(t) = \hbar \sum_{kl} c_{m1kl} b_{kl} + \hbar\Delta b_{m1} + \hbar\Omega a_{m1}, \tag{S19}$$

$$i\hbar \dot{b}_{mn}(t) = \hbar \sum_{kl} c_{mnkl} b_{kl}, \qquad \text{for } n > 1. \tag{S20}$$

In this form, all the important dynamics are captured, and the localized component can be slow relative to the mediating component.

## III. HOPPING KERNELS WITH A LATTICE

Suppose the mediating channel has a much faster time scale, so the adiabatic elimination is the same as setting $\dot{b}_{mn} = 0$. Therefore, the hopping kernel can be found by solving $b_{m1}$ in the following self-consistently equation by having $a_{m1} = 1$ at the center:

$$0 = \sum_{kl} c_{m1kl} b_{kl} + \Omega a_{m1} + \Delta b_{m1}, \tag{S21}$$

$$0 = \sum_{kl} c_{mnkl} b_{kl} \qquad \text{for } n > 1,$$

This is the discrete analogue of finding the continuous hopping kernel $G_D(\mathbf{r})$ as described in the main text by setting $\psi_1(\mathbf{r}) = \delta(\mathbf{r})$, except the interconversion only happens in certain regions. The effective conversion regions have a length scale $2\ell$ of the localized wavefunction, in each lattice unit with length $d$. Therefore, it is expected that the solution $G_{ij}$ takes a similar form as the continuous system with an effective scaling $\Delta \to \Delta_{eff} = (2\ell/d)^D \Delta$. Hence, the solution is $b_{i1} = \frac{\Omega}{\Delta} G_{ij} * a_{j1}$. Substituting back into the first component, the hopping strength becomes

$$P = \hbar \frac{\Omega^2}{\Delta}, \tag{S22}$$

the same as the continuous system, and $G_{ij}$ takes the same form as in the Table 1 in the main text. The characteristic hopping radius is

$$R = C_D \left(\frac{d}{2\ell}\right)^{\frac{D}{2}} \sqrt{\frac{\kappa}{\Delta}}, \tag{S23}$$

where $D$ is the dimension and $C_D$ is a constant.

The results above can be verified numerically. This requires a method to find the hopping kernel in a periodic lattice self-consistently. Here, we solve the corresponding time dependent equation of Eq. (S21) and the solution is given by the equilibrium state. Hence, Eq. (S21) with the time splitting method becomes

$$\dot{b}_{m1}(t) = - \Omega a_{m1} - \Delta b_{m1}, \tag{S24}$$

$$\dot{\tilde{\psi}}_2(\mathbf{q}, t) = - \frac{\hbar q^2}{2m} \tilde{\psi}_2, \tag{S25}$$

for the conversion step and propagation step respectively, so the basis is changed between each step. $\tilde{\psi}_2(\mathbf{q}, t)$ is the wavefunction in Fourier space. The hopping kernel $G_{ij}$ is the same as the equilibrium solution $b^*_{i1}$ with $G_{mj} \sim b^*_{m1}$, if the system is set to $a_{m1} = \delta_{j1}$, where $j$ is the source lattice site (chosen to be the center of the lattice). For simplicity, Gaussian approximation is used to approximate the lowest band Wannier function as

$$w_{m1}(\mathbf{r}) = \phi(\mathbf{r} - \mathbf{r}_m) \sim e^{-\frac{\pi|\mathbf{r}-\mathbf{r}_m|^2}{2\ell^2}}, \tag{S26}$$

which is a good approximation when $\ell \ll d$, such as deep sinusoidal trap, where $\mathbf{r}_m$ is the center of the Gaussian. The transformation between real space and Wannier basis are given by

$$b_{m1} = \langle w_{m1}(\mathbf{r}) | \psi_2(\mathbf{r}) \rangle = \int_V d^3\mathbf{r} \phi(\mathbf{r}) \psi_2(\mathbf{r}), \tag{S27}$$

where $V$ is the lattice volume around the lattice minimum $[-d/2, d/2]^D$ with finite cutoff of lattice spacing $d$.

The numerical results fit perfectly for the kernel $G_D(r)$ in both 1D and 2D as shown in Fig. S1. Moreover, the predicted hopping radius $R$ fit perfectly with Eq. (S23) when the Gaussian $\ell \ll d$ is sufficiently narrow such that the approximation is good. Both of these fitting give the constant $C_D \approx 1$.

## IV. CHIMERA PATTERNS IN THE NLHM

### A. Other initial conditions in 1D and the effect of hopping strength

As shown in Fig. 2 in the main text, the random phase initial condition with uniform amplitude gives a chimera pattern that has a low local order parameter $|\mathcal{O}|$ at the center. A lower $|\mathcal{O}|$ can be obtained by using the initial condition that has both random phase and random amplitude as given by Fig. S2(c) and S2(d). The magnitude of $|\mathcal{O}|$ is even lower than Fig. 2 in the main text, implying that the state is even less coherent. Suppose the phase of the region $-R < x < R$ is completely random while constant outside, then the order parameter gives the $|\mathcal{O}_{i=0}| = \sum_j G_{ij} e^{i\theta_j} \approx (1/R) \int_R^\infty e^{-z/R} dz = e^{-1}$, which is roughly the value for the random phase initial condition at $t = 0$ at the center. As shown in Fig. S2(e), $|\mathcal{O}_{i=0}| \approx e^{-1}$ at $t = 400$, that means the phase are still completely random near the center after some times.

For a sufficiently small hopping strenght $P$, the oscillators are spatially decoupled while the local phase and amplitude are still strongly coupled as shown in Eq. (3) in the main text. Simulations suggest that stable chimera patterns can still exist even for very small $P$ and in the strong hopping regime as shown in Fig. S3. In the regime with very small $P$, the amplitude fluctuates around the initial amplitude by a small amount. On the other hand, in the strong hopping regime, the chimera pattern with the random phase initial condition still exist, but the amplitude fluctuates a lot larger. In general, a chimera pattern can be more difficult to emerge from a regular pattern with strong $P$. For example, with the same vortex initial condition in Fig. 3 in the main text, no incoherence region has formed in the strong hopping regime. Nevertheless, with the 2D vortex initial condition, the incoherence core can still emerge and it is the one of the main difference observed between 1D and 2D.

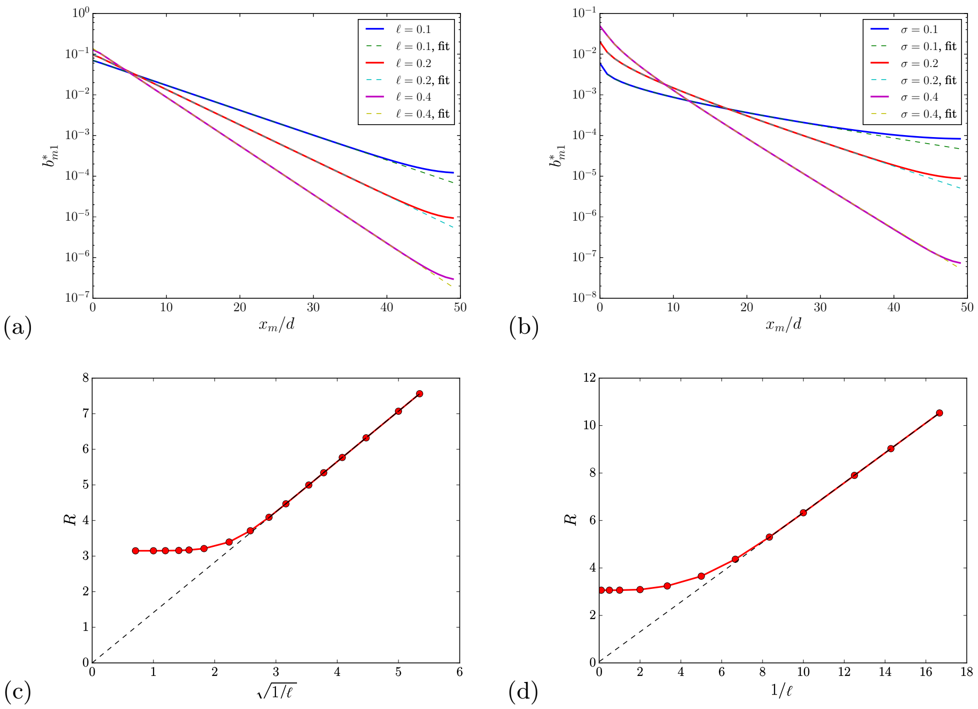


Figure S1: Discrete hopping kernel $G_{ij}$. (a) Comparing the numerical kernel $b^*_{m1}$ with exponential fitting in 1D. $\kappa = 100$, $\Omega = 10$, and $\Delta = 10$. (b) Comparing $b^*_{m1}$ with $K_0$ fitting in 2D. (c) The scaling of the hopping radius $R \sim \ell^{-1/2}$ in 1D versus a length scale $\ell$. (d) The scaling of the hopping radius $R \sim \ell^{-1}$ in 2D versus $\ell$.

### B. Spiral wave and vortex-like initial condition

The snaphots at different time with the spiral initial condition used in the main text is given in Fig. S4 (and an animation). As shown in the figure, the incoherent core is spontaneously formed near the spatial phase singularity. This dynamic pattern is essentially invariant under the scaling with fixed $R/L$ as shown in Fig. S5, in which both system size $L$ and hopping radius $R$ are four times larger. The core has locally incoherent phase, while the dynamics outside the core are locally coherent. This pattern can persist over a long time once formed. So if we start with the random phase core, the chimera pattern can also persist over a long time, as shown in Fig. S6.

Similar to the spiral wave initial condition, the chimera patterns can form with a vortex-like initial condition, as shown in Figs. S6 and Figs. S7. The snaphots of the dynamic of a vortex dip at the center used in the main text is given in Fig. S7, which can be compared with Fig. S4.

The dynamics are very different when the hopping range becomes small $R \sim d$. With only nearest-neighbors hopping, the system becomes the discrete Gross-Pitaevskii equation. The difference is very clear when the system is started from the same random core with nearest-neighbor hopping as shown in Fig. S8. The random phase near the core is a localized perturbation that propagates outward like a wave and interferes with itself. In this case, no localized chimera core can be observed.

### C. Noise

The behaviour of the core is very different from the background. In particular, the dynamics of the core are very sensitive to small fluctuations or noises. For example, we can evolve the system backward in time and expect it to go back to the initial spiral, as shown in S9a, if the system starts from the state in Fig. S4f. The sensitivity of the core region can be tested by adding a small single-shot noise

$$a_i \rightarrow a_i + \chi_{\text{noise}}\xi_i, \tag{S28}$$

where the noise is Gaussian with $\langle \xi_i \rangle = 0$, $\langle \text{Re}(\xi_i)\text{Re}(\xi_{i'}) \rangle = \delta_{i,i'}$, and $\langle \text{Im}(\xi_i)\text{Im}(\xi_{i'}) \rangle = \delta_{i,i'}$, with amplitude $\chi_{\text{noise}}$. This noise can be added to the state in Fig. S4f as a perturbation before the backward propagation. As shown in Fig.

S9b and S9c, the system cannot go back to the spiral even with a noise as low as $\chi_{\text{noise}} = 10^{-11}$, as compared to the order $\mathcal{O}(1)$ of the amplitude and phase. This suggests that the core region is very sensitive to the initial condition. This is in stark contrast to with the behaviour of the coherent background, which can go back to the same local states as in the noiseless case.

### D. Loss

The nonlinear particle loss can be modelled by the replacing $U \to U - iU_{\text{loss}}$. Therefore, the dynamic equation with loss is

$$i\hbar\dot{a}_i = (U - iU_{\text{loss}})|a_i|^2 a_i - P\sum_j G_{ij}a_j. \tag{S29}$$

The particle loss can be calculated by the time-derivative of the total number of particles:

$$\frac{dN}{dt} = \frac{d}{dt}\sum_i |a_i|^2 = \sum_i (\dot{a}_i^* a_i + a_i^* \dot{a}_i) = \sum_i -\left(2\frac{U_{\text{loss}}}{\hbar}\right)|a_i|^4, \tag{S30}$$

where the dynamic equations for $\dot{a}_i$ and $\dot{a}_i^*$ are substituted above. Suppose all lattice sites have the same number of particles $|a_i|^2 = n$, then the equation becomes

$$\frac{dn}{dt} = -\left(2\frac{U_{\text{loss}}}{\hbar}\right)n^2. \tag{S31}$$

Let $\chi = 2U_{loss}/\hbar$ and then by solving the equation, we have

$$n(t) = \frac{1}{n_0^{-1} + \chi t}, \tag{S32}$$

where $n_0$ is the initial number of particles per site. Suppose $n_0$ is very large, then the time for half of the particle to become lost is $\chi t = 2$ or $U_{loss}t/\hbar = 1$. As shown by the order parameter $|\mathcal{O}_i|$ in Fig. S10, the system becomes more and more coherent over time with loss. Moreover, as shown in Fig. S11, the phase of the chimera patterns changes slightly compared to the lossless case on timescales where many more than half of the particles remain in the system.

[S1] D. Witthaut and M. Timme, Phys. Rev. E **90**, 032917 (2014).
[S2] Q. Thommen, J. C. Garreau, and V. Zehnlé, Phys. Rev. Lett. **91**, 210405 (2003).
[S3] E. Brion, L. H. Pedersen, and K. Mølmer, J. Phys. A: Math. Theor. **40**, 1033 (2007).
[S4] D. Jaksch, C. Bruder, J. I. Cirac, C. W. Gardiner, and P. Zoller, Phys. Rev. Lett. **81**, 3108 (1998).
[S5] O. Morsch and M. Oberthaler, Rev. Mod. Phys. **78**, 179 (2006).

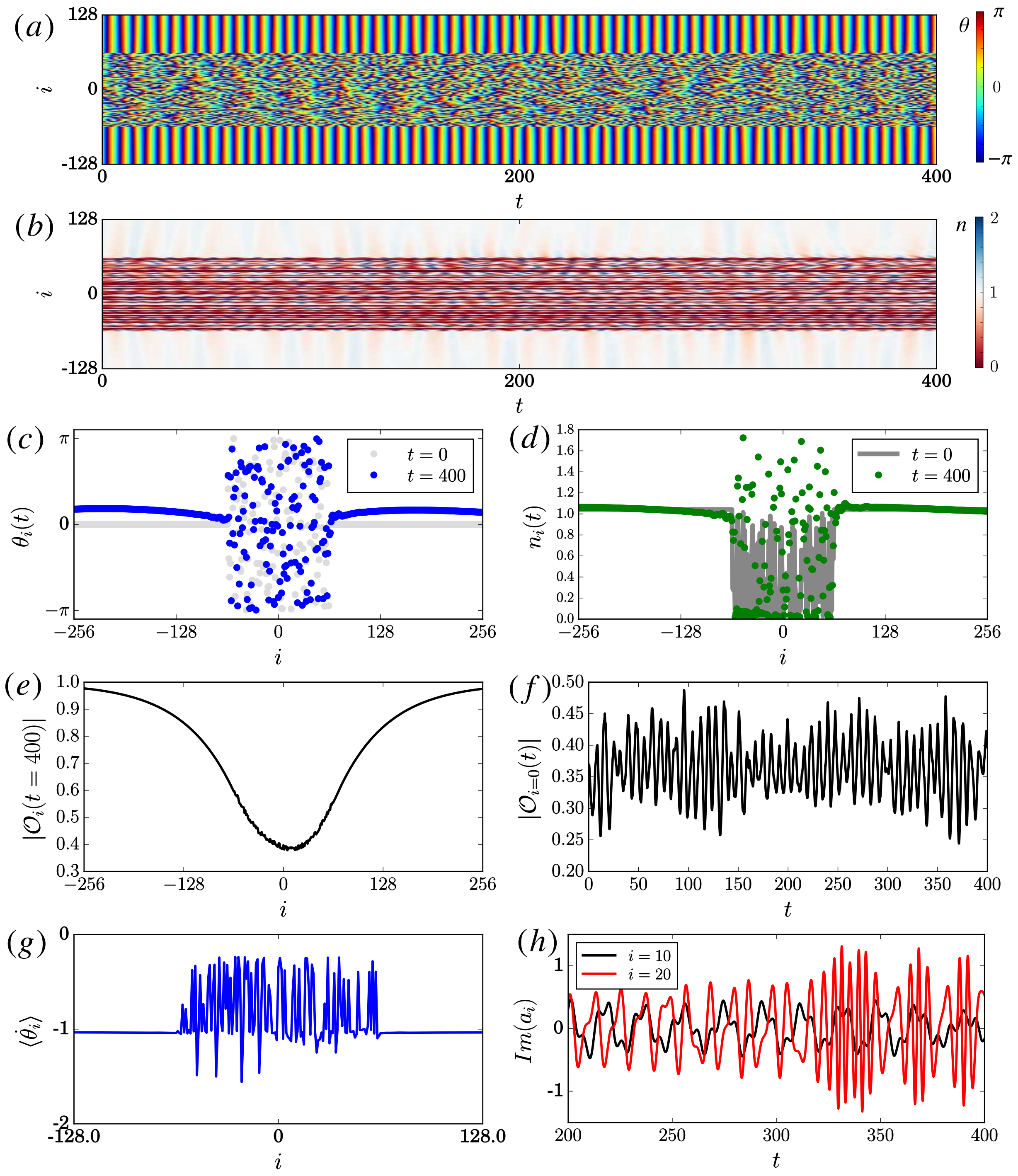


Figure S2: Similar to Fig. 2 in the main text, but with initial random phase and random amplitude as given in subfigure (c) and (d). Note that $|\mathcal{O}_{i=0}| \approx e^{-1}$ for a region to be fully incoherent as calculated in text. Same parameter as in Fig. 2, but with $N = \sum_i |a_i|^2 = L$.

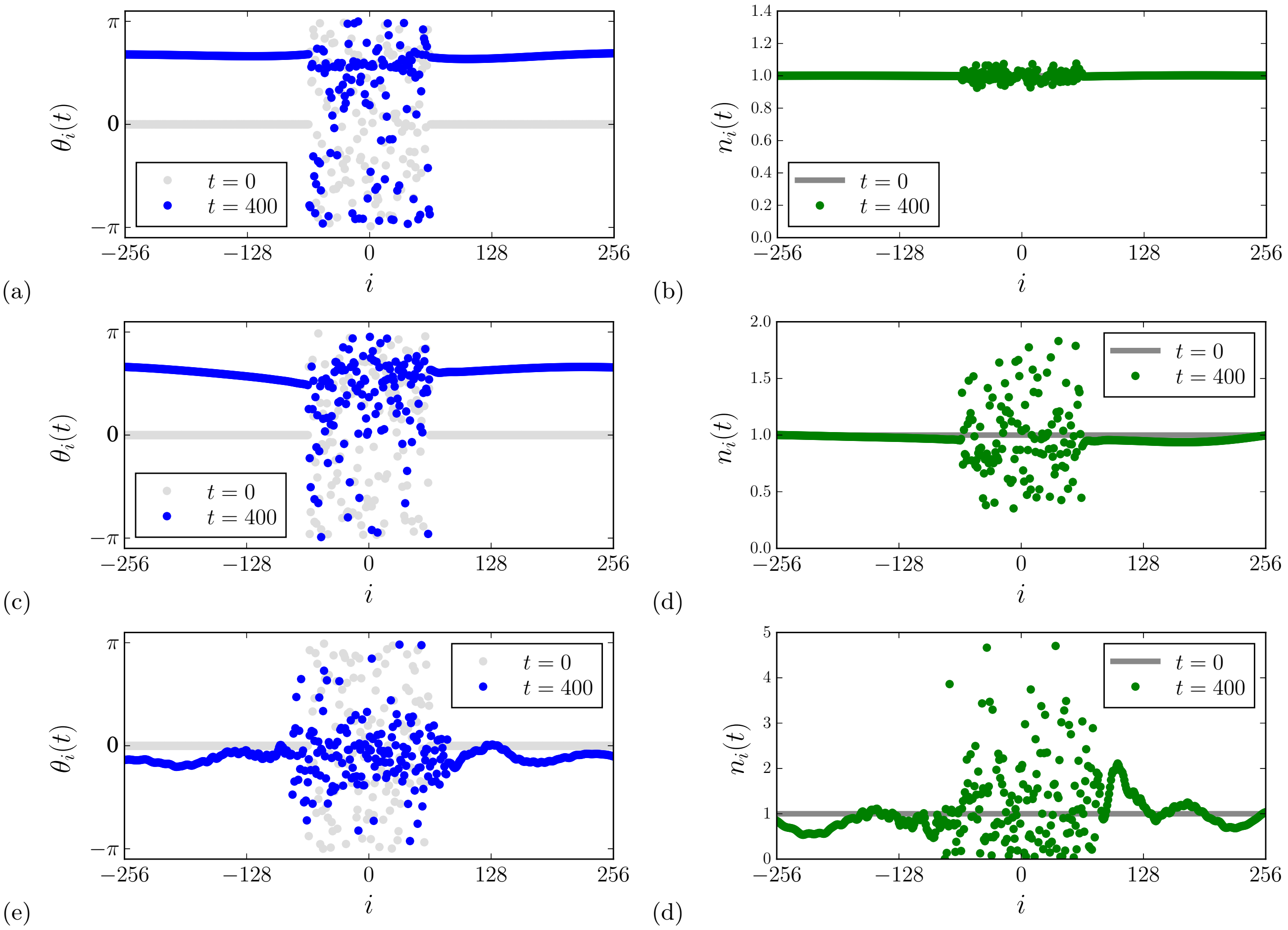


Figure S3: Effects of different hopping strenght $P$. (a,b) $P = 10^{-4}$, (c,d) $P = 0.1$, (e,f) $P = 5$.

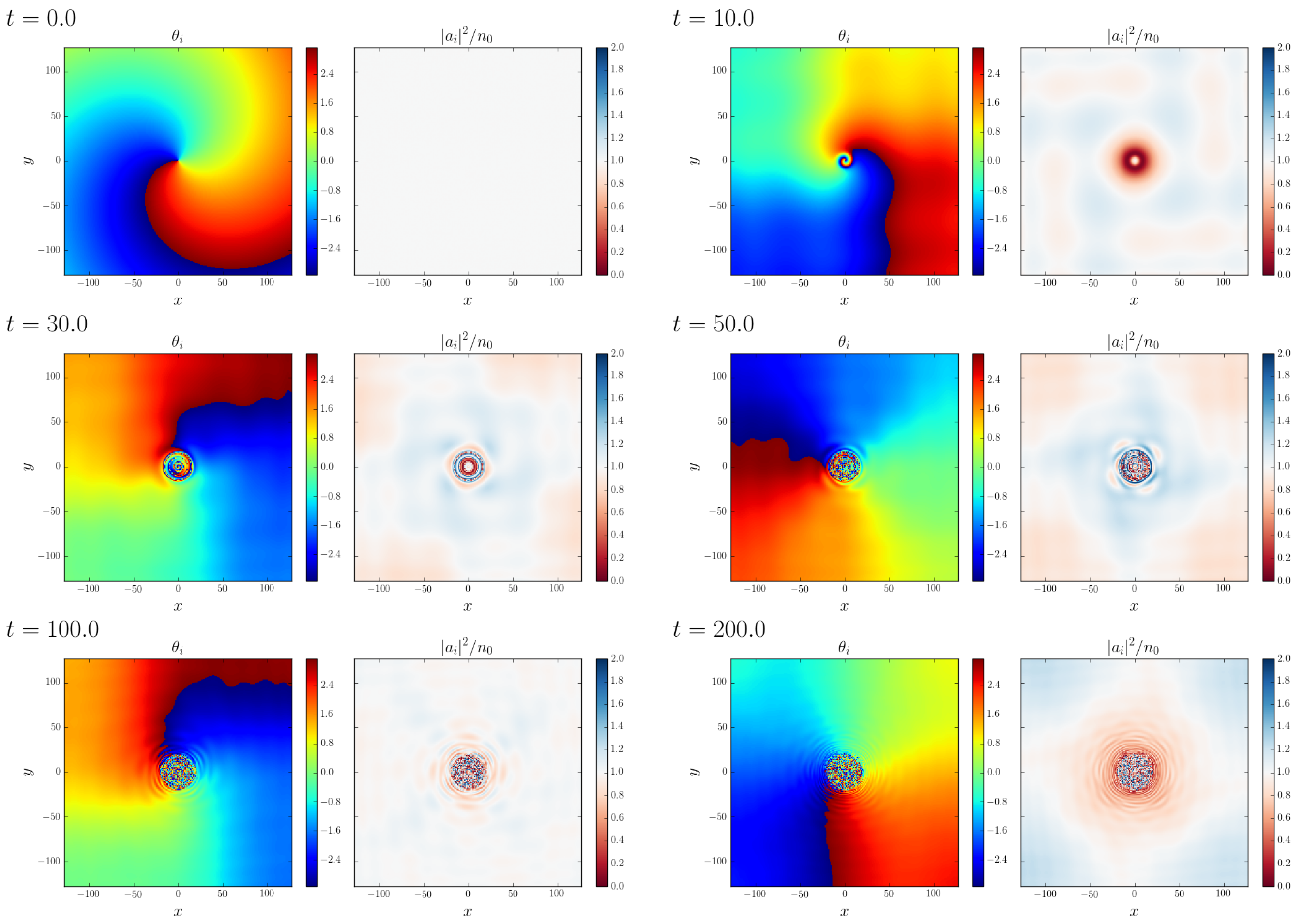


Figure S4: Time evolution of the initial spiral with $k_s = 0.01$. We used nonlocal hopping $P/(Un_0) = 0.5$ and $R = 16$ in a system with a lattice size of $L = 256$.

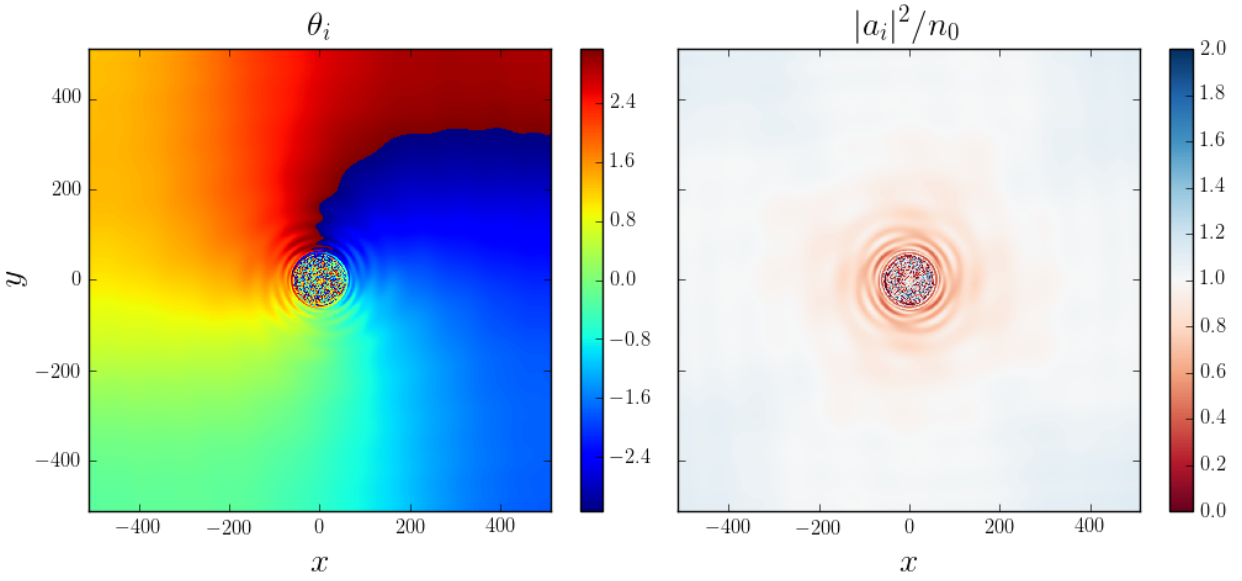


Figure S5: Similar to $t = 100$ Fig. S4 but in a larger system with initial spiral $k_s = 0.0025$. We use nonlocal hopping $P/(Un_0) = 0.5$ and $R = 64$ in a system with a lattice size of $L = 1024$. This can be compared with Fig. S4e.

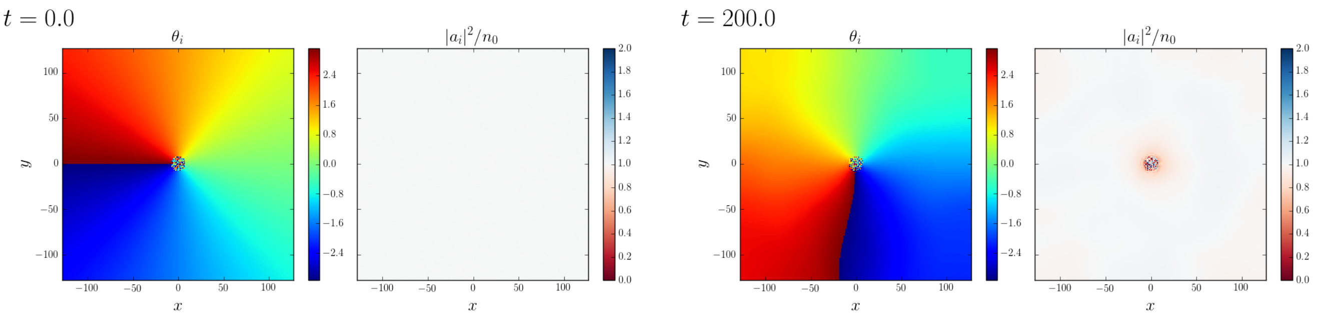


Figure S6: Time evolution of an initial random phase core with radius $R_{core} = 8$ and uniform amplitude. $P/(Un_0) = 0.5$, $R = 8$, and $L = 256$.

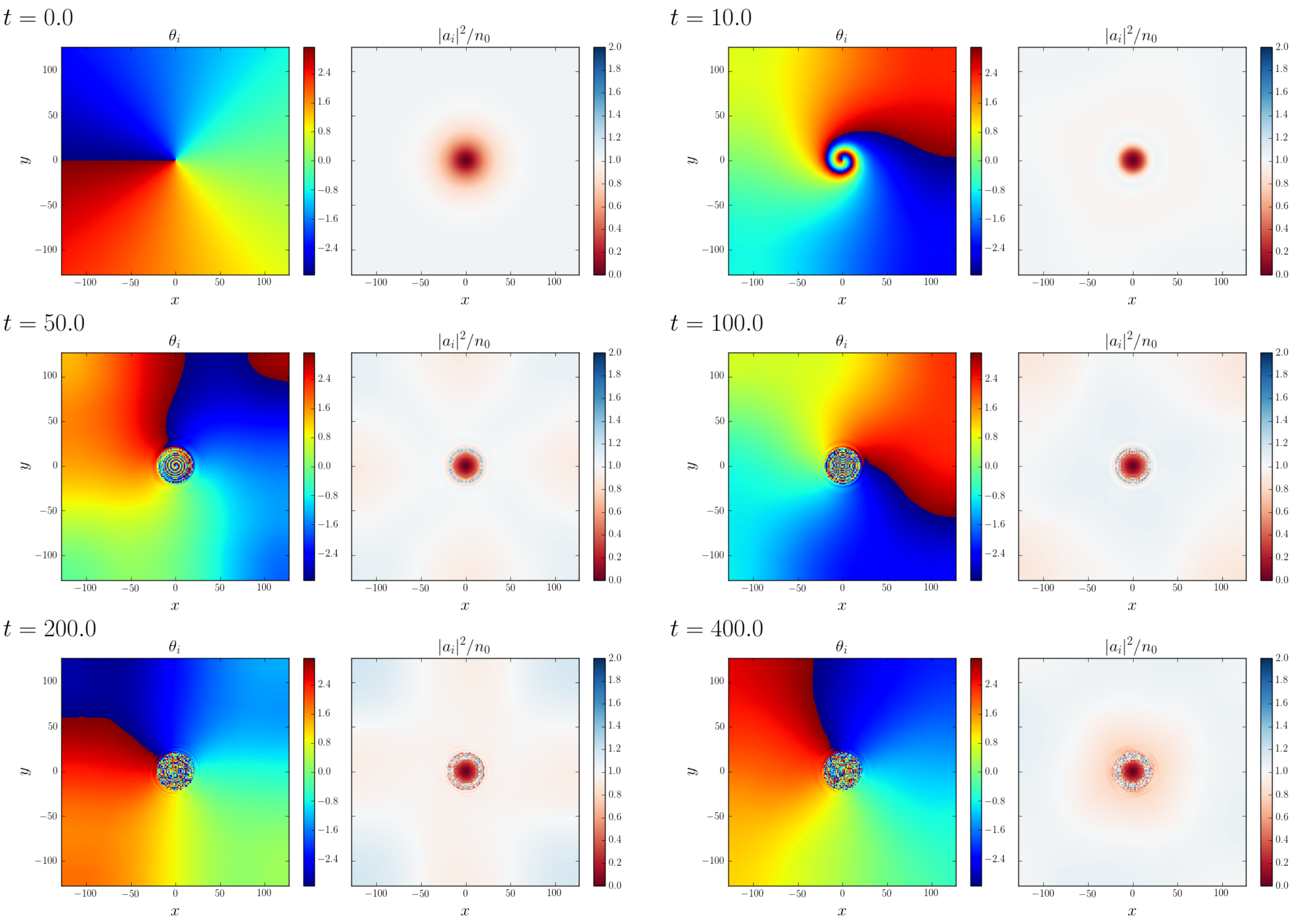


Figure S7: Time evolution of an initial vortex with $R_{vortex} = 16$. We used nonlocal hopping $P/(Un_0) = 0.1$ and $R = 16$ in a system with a lattice size of $L = 256$.

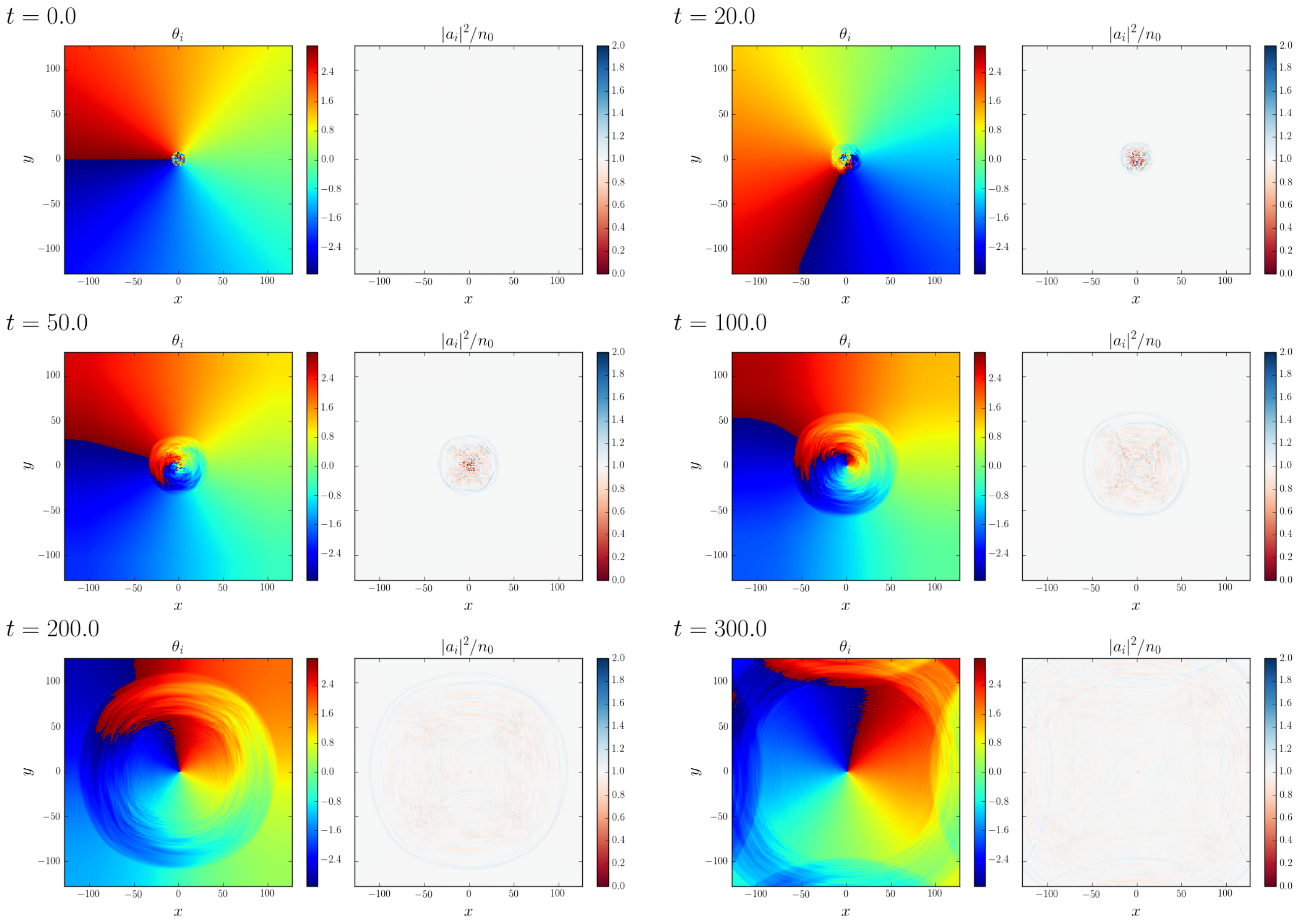


Figure S8: Time evolution of an initial random core with radius $R_{core} = 8$ with only nearest-neighbor hopping. $P/(Un_0) = 0.5$ and $L = 256$.

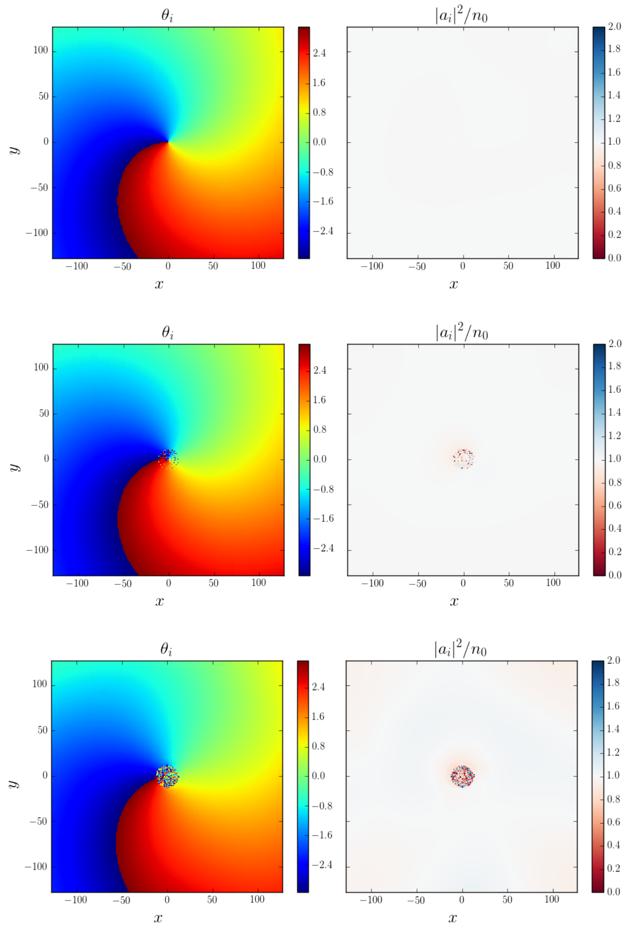

(a)

(b)

(c)

Figure S9: Backward time propagation for the duration $t = 200$ using the state $t = 200$ in Fig. S4 as the initial condition. (a) No noise, (b) $\chi_{noise} = 10^{-11}$, (c) $\chi_{noise} = 10^{-10}$, where the single-shot noises are add before the backward propagation. The noise added is very tiny $\chi_{noise}/|a_i| \sim \chi_{noise}$ since $|a_i| \sim 1$ is used.

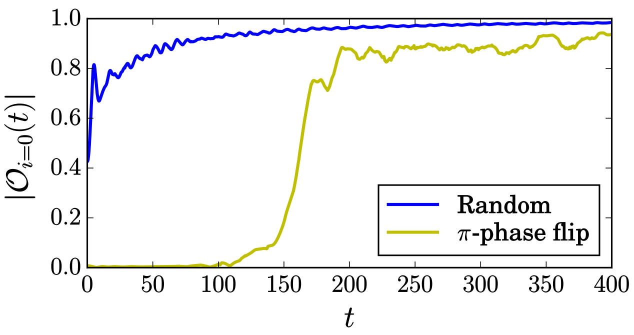


Figure S10: NLHM in 1D under nonlinear loss for the two initial conditions in Fig. 2 and Fig. 3 in the main text. The loss used is $U \to U - iU_{loss}$ where $U_{loss} = 0.01$. The center becomes synchronized with the system when $\mathcal{O} \sim 1$.

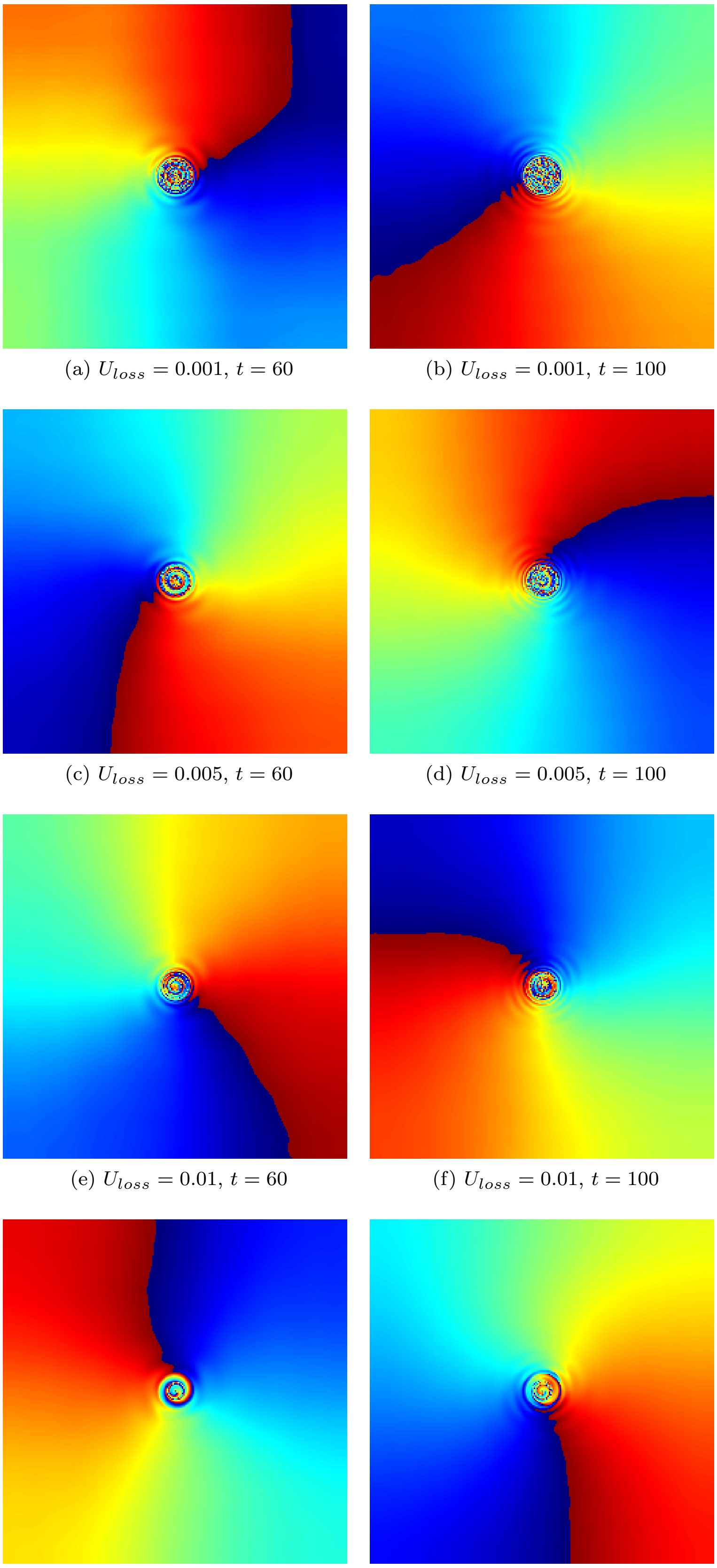

(a) $U_{loss} = 0.001$, $t = 60$

(b) $U_{loss} = 0.001$, $t = 100$

(c) $U_{loss} = 0.005$, $t = 60$

(d) $U_{loss} = 0.005$, $t = 100$

(e) $U_{loss} = 0.01$, $t = 60$

(f) $U_{loss} = 0.01$, $t = 100$

(g) $U_{loss} = 0.02$, $t = 60$

(h) $U_{loss} = 0.02$, $t = 100$

Figure S11: Similar to Fig. S4 with loss $U_{loss} = 0.001, 0.005, 0.01, 0.02$ from top to bottom. (left) $t = 60$, (right) $t = 100$. Parameters: $U = 1$, $n_0 = 1$, $P = 0.5$, $R = 16$, and $L = 256$.
